# Lipophorin receptors regulate mushroom body development and complex behaviors in *Drosophila*

**DOI:** 10.1186/s12915-022-01393-1

**Published:** 2022-09-07

**Authors:** Francisca Rojo-Cortés, Nicolás Fuenzalida-Uribe, Victoria Tapia-Valladares, Candy B. Roa, Sergio Hidalgo, María-Constanza González-Ramírez, Carlos Oliva, Jorge M. Campusano, María-Paz Marzolo

**Affiliations:** 1 Laboratorio de Tráfico Intracelular y Señalización, Departamento de Biología Celular y Molecular, Facultad de Ciencias Biológicas, Pontificia Universidad Católica de Chile, Santiago, Chile; 2 Laboratorio Neurogenética de la Conducta, Departamento de Biología Celular y Molecular, Facultad de Ciencias Biológicas, Pontificia Universidad Católica de Chile, Santiago, Chile; 3 Department of Biology, University of Puerto Rico, Rio Piedras, San Juan Puerto Rico; 4 Department of Entomology and Nematology, College of Agricultural and Environmental Sciences, University of California, Davis, USA; 5 Laboratorio Neurodesarrollo, Departamento de Biología Celular y Molecular, Facultad de Ciencias Biológicas, Pontificia Universidad Católica de Chile, Santiago, Chile

**Keywords:** Lipophorin receptor, Lipoprotein receptor, Mushroom bodies, Reelin, Dab, Neuronal development, Neurite morphogenesis

## Abstract

**Background:**

*Drosophila melanogaster *lipophorin receptors (LpRs), LpR1 and LpR2, are members of the LDLR family known to mediate lipid uptake in a range of organisms from *Drosophila* to humans. The vertebrate orthologs of LpRs, ApoER2 and VLDL-R, function as receptors of a glycoprotein involved in development of the central nervous system, Reelin, which is not present in flies. ApoER2 and VLDL-R are associated with the development and function of the hippocampus and cerebral cortex, important association areas in the mammalian brain, as well as with neurodevelopmental and neurodegenerative disorders linked to those regions. It is currently unknown whether LpRs play similar roles in the *Drosophila* brain.

**Results:**

We report that LpR-deficient flies exhibit impaired olfactory memory and sleep patterns, which seem to reflect anatomical defects found in a critical brain association area, the mushroom bodies (MB). Moreover, cultured MB neurons respond to mammalian Reelin by increasing the complexity of their neurite arborization. This effect depends on LpRs and Dab, the Drosophila ortholog of the Reelin signaling adaptor protein Dab1. In vitro, two of the long isoforms of LpRs allow the internalization of Reelin, suggesting that *Drosophila* LpRs interact with human Reelin to induce downstream cellular events.

**Conclusions:**

These findings demonstrate that LpRs contribute to MB development and function, supporting the existence of a LpR-dependent signaling in *Drosophila*, and advance our understanding of the molecular factors functioning in neural systems to generate complex behaviors in this model. Our results further emphasize the importance of *Drosophila* as a model to investigate the alterations in specific genes contributing to neural disorders.

**Supplementary Information:**

The online version contains supplementary material available at 10.1186/s12915-022-01393-1.

## Background

The low-density lipoprotein receptor (LDL-R) family is an ancient protein family conserved throughout evolution [1, 2]. In mammals, these proteins include two groups of membrane receptors, one of seven members including LDL-R, LRP-1, LRP-1B, LRP-2/megalin, LRP-8/ApoER2, VLDL-R, and MEGF7 and the second more distant group including SorLA/LR11 and the Wnt signaling receptors LRP-5 and LRP-6 [3]. The LDL-R family is widely known because of its role in lipoprotein endocytosis, controlling the systemic homeostasis of cholesterol by binding to apolipoprotein E (ApoE) and apolipoprotein B (ApoB) [4, 5] as well for their signaling properties [3, 6, 7]. *Drosophila melanogaster* express Arrow [8–10], the ortholog of LRP-5/6, Dmel/mgl the ortholog of mammalian LRP2/megalin involved in the control of melanization of the cuticle [11], and lipophorin receptors (LpRs) 1 and 2 [12–14]. LpR1 and LpR2 have been linked to lipid uptake in the context of oogenesis and neuronal activity, which is consistent with the known roles of several LDL-R family members in vertebrates [13, 15–17]. Lipophorins are the invertebrate’s molecular entity equivalent to lipoproteins in vertebrates and bind LpRs through their protein component (apolipophorin) [12]. In contrast to lipoproteins, which are destined to degradation in lysosomes, lipophorins are recycled with LpRs, after lipid unloading and uploading, similar to transferrin [1, 2].

Structurally, LpRs consist of seven to eight LA modules, three EGF modules, a β-propeller, an O-glycosylation site near the transmembrane domain, and a cytoplasmic tail [13]. Six isoforms for LpR1 and five for LpR2 have been described in *Drosophila*. These are generated by alternative use of two distinct promoters, proximal or distal [13]. The main consequence of the use of the proximal promoter is that the resulting transcript lacks exons 1 to 3, which encode for a non-conserved N-terminal region and the first LA module [13]. Thus, the isoforms transcribed from the proximal promoter are also known as the “short” ones, while those transcribed from the distal promoter are known as the “long” isoforms [13, 16, 18].

Only a subset of LpR isoforms mediate lipid uptake [13, 16–18]. Several reports link LpRs to functions beyond lipid metabolism. The first one showed that *Drosophila* LpR1 increases the immune response by modulating the uptake and degradation of Necrotic Serpin, a protein that controls the innate response to gram-negative infection in insects [14]. A second study showed that LpRs play a role in dendrite morphogenesis in larval ventral lateral neurons (LNv), a neuronal group that is part of the circuit that controls circadian rhythms in *Drosophila* [19]. Moreover, in the case of LpR1, this effect depends on the short isoforms. Specifically, it was reported that knocking down a specific LpR1 short isoform (LpR1G) or LpR2 in LNv reduces activity-dependent dendrite arborization [18, 19]. Since a genome-wide RNAi screen in cultured neurons from *D. melanogaster* embryos identified LpR2 as a gene implicated in neuronal development [20] and given the fact that LpR1 and LpR2 are widely expressed in the *Drosophila* larval brain [18, 19], it is possible to propose that LpRs could contribute to the development, maturation, and operation of several fly brain regions.

*Drosophila* LpR1 and LpR2 share with vertebrates LDL-R, VLDL-R, and ApoER2, motifs involved in intracellular trafficking and signaling, like the NPxY [2, 13]. In this regard, besides their role as lipoprotein receptors, VLDL-R and ApoER2 serve as receptors for the secreted glycoprotein Reelin, an extracellular ligand with essential roles in the central nervous system (CNS) [21–24]. Reelin functions include neuronal polarization and migration in critical association areas in the brain, such as the cerebral cortex, hippocampus, and cerebellum. In adult animals, Reelin is also implicated in synaptic plasticity, learning, and memory [22, 23, 25–31].

Here, we decided to advance on the characterization of the expression and function of LpRs in the fly brain. First, we showed that *Drosophila* mutants for LpR1 and LpR2 exhibit olfactory memory and sleep architecture deficits. Since these behaviors depend on a crucial associative region in the fly brain, the mushroom bodies (MB), we assessed whether LpR expression has any consequence on the structure of this fly brain area. Our data show relevant alterations in the MB organization of fly mutants for LpRs and also of animals where an RNAi for these receptors is directed to this brain region. Surprisingly, *Drosophila* MB neurons in primary culture were responsive to vertebrate Reelin by increasing their neurite tree’s complexity, as vertebrate neurons do. This effect depends on LpR1 and LpR2. Also, we show that cells bearing long isoforms of LpR1 and LpR2 internalize Reelin. In line with the idea of a Reelin-induced LpR-dependent response in flies, we described that MB organization and function, and the response of cultured MB neurons to Reelin, are affected by the absence of the cytoplasmic protein Dab, the fly homolog of Disabled 1 (Dab1) [32–34], which is the first component of the Reelin intracellular signaling cascade [35]. Overall, our results show, for the first time, that LpR1 and LpR2 contribute to *Drosophila* MB organization and function. Besides, our data suggest that both receptors would participate in a novel signaling cascade involving Dab, whose homolog, Dab1 in vertebrates, can be activated by Reelin.

## Results

### Fly mutants for lipophorin receptors exhibit impaired behaviors associated with MB function

**Fig. 1:**
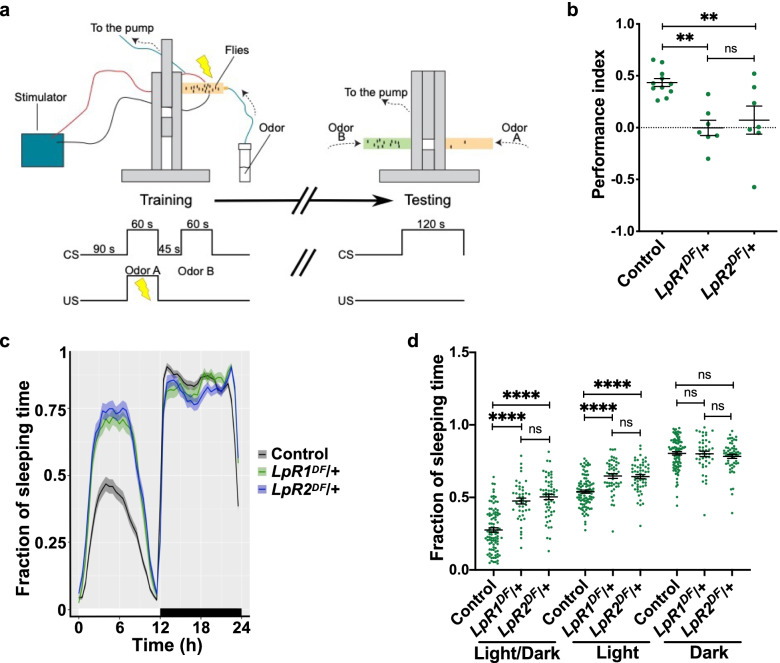
Impairment in MB-associated behaviors in LpR1 and LpR2 mutants. **a** Scheme of the setup and protocol for aversive olfactory memory assay. Flies were allowed to familiarize themselves with the training apparatus for 90 s. Then, flies were exposed to an odorant (conditioned stimulus, CS +) over 60 s, while they received electric shocks (unconditioned stimulus, US) (left panel). After this, flies were allowed to rest for 45 s in the presence of fresh air. Afterward, flies were exposed to a different odorant for 60 s, which was not paired to electric shocks. One hour after this training, memory was evaluated. Memory test (right panel) consists of having the flies in the center of the apparatus where they are exposed to the two odorants (each in one arm) for 120 s. In that period, flies are allowed to choose between them. **b** Performance index for olfactory memory in control animals and mutant flies for LpR1 (*LpR1*^*DF*^/ +) or LpR2 (*LpR2*^*DF*^/ +). *n* = seven experiments carried out in independent groups of flies. One-way ANOVA followed by Dunnett’s test, *p* = 0.0011. ** indicates *p* < 0.01, “ns” not significant. **c** Sleep profile throughout the day in flies mutant for LpR1 or LpR2. Above the *x*-axis, the white bar represents the hours of the day when flies were exposed to light; the black bar represents the hours when flies were exposed to darkness. **d** Fraction of sleeping time in flies mutants for LpR1 and LpR2. Two-way ANOVA shows that light condition and genotype factors, and also interaction between these factors, play a role in results (*p* < 0.0001 for each analysis); Tukey post-test; *, **, and ****, indicates *p* < 0.05, *p* < 0.01, and *p* < 0.0001 between conditions. ns, not significant. Data in **c** and **d**, from *n* = 43–95 flies studied per genotype in two independent experiments. In **b**–**d**, data expressed as mean ± SEM

In order to characterize the role of LpRs in the *Drosophila* brain, we first asked whether mutants for these receptors exhibit any alteration in one of the most studied behaviors in flies, aversive olfactory memory. We used a mutant for each LpR, generated by deleting a segment of the encoding genes [13]. These tools were validated through qPCR (Additional file 1: Fig. S1a-b). We carried out a protocol to generate mid-term aversive olfactory memories, as explained in the “Methods” section (Fig. 1a). Briefly, flies were exposed to two different odorants sequentially, but only the first odorant was paired to electric shocks. One hour after finishing the training protocol, the flies were allowed to choose between the two odorants. In control animals, the olfactory memory measured as performance index reached a magnitude of 0.44 ± 0.04, consistent with the idea that the training protocol generates new olfactory memories. Remarkably, the performance index recorded in the LpR1 and LpR2 mutants suggests that no memories were generated in these flies (0.00 ± 0.07 and 0.07 ± 0.14, respectively; *p* < 0.01 as compared to controls; Additional file 1: Fig. 1b). Additionally, as experimental controls, we evaluated the mutants’ ability to respond to the conditioning stimulus (electric shocks; Additional file 1: Fig S2a) and the odorants used (Additional file 1: Fig S2b-c). Interestingly, LpR1 mutants display a higher olfactory acuity toward one of the odorants used (Additional file 1: Fig S2b), which is one of the reasons we carried out the training protocol using either odorant as a conditioned stimulus for different experiments.

We then assessed circadian rhythms in LpR mutants while under a light–dark cycle of 12–12 h [36, 37]. Interestingly, the observation of actograms supports the idea that flies mutant for LpR1 and LpR2 exhibit increased sleeping time compared with control flies, particularly during the light phase (Fig. 1c, d). A summary of data collected over 6 days evidenced higher sleep time in both mutant strains during the light phase (Fig. 1d). Moreover, the expression of RNAi against LpR1 or LpR2 into MB neurons also increased the fraction of time sleeping during the light phase (Additional file 1: Fig. S3) compared to control animals. The RNAis were validated by qPCR using the pan-neuronal driver Elav (Additional file 1: Fig. S1c-d).

Altogether, these results support the idea that tampering with the expression of LpRs has functional consequences reflected by impairment in memory formation and the regulation of sleep patterns.

### Lipophorin receptors contribute to adult mushroom body organization

The MB is a well-characterized *Drosophila* structure located in each side of the brain, linked to crucial associative fly behaviors, including olfactory learning and memory, sleep, and locomotion [38–42]. Therefore, we decided to study whether alterations in olfactory memory and sleep homeostasis in mutants for LpRs are associated with changes in MB anatomy.

**Fig. 2:**
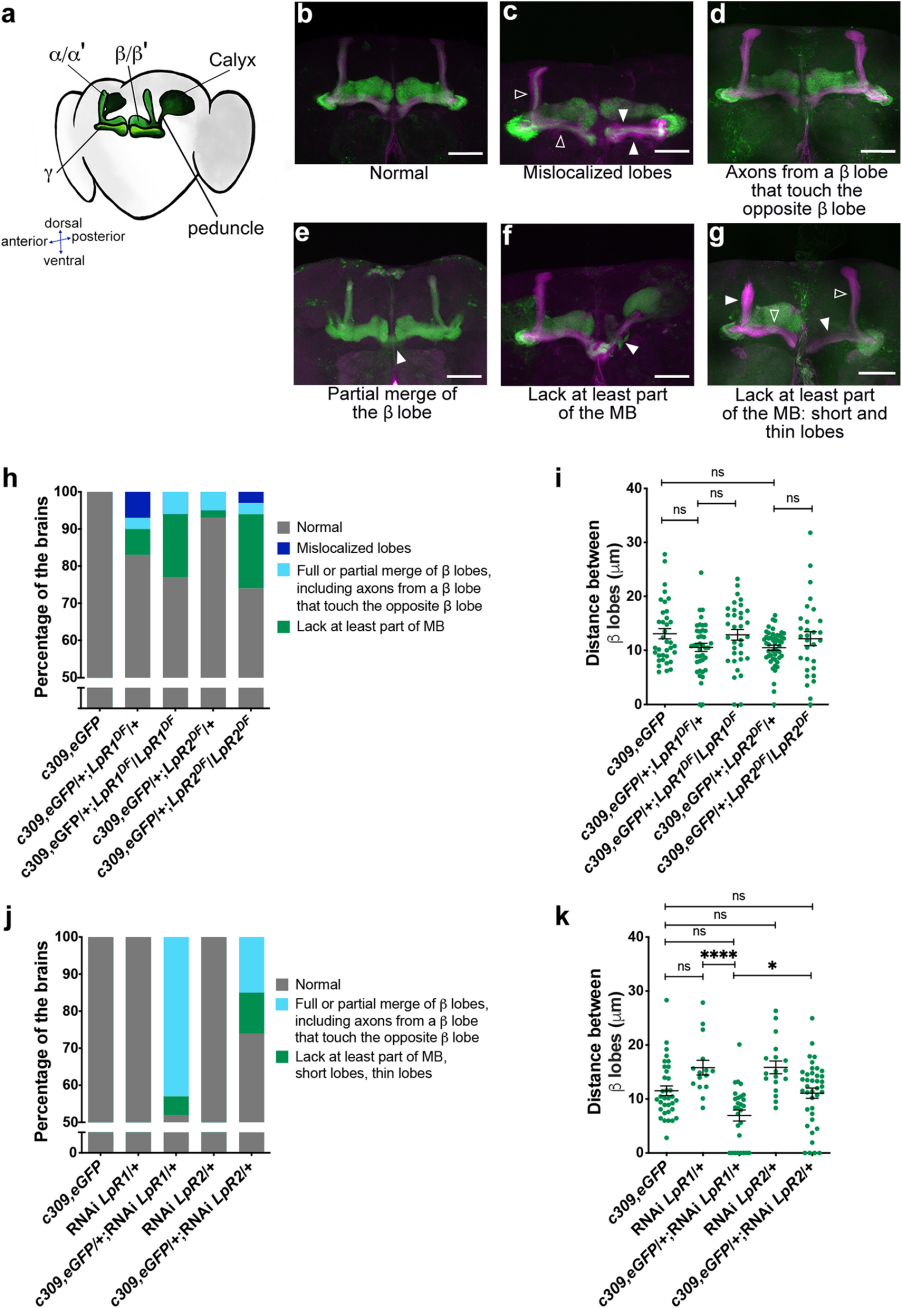
Decreasing expression of LpR1 or LpR2 in MB results in anatomical defects.** a** Scheme of the normal organization of the MB in the adult fly brain. MB subregions are identified. **b–g** Representative images of phenotypes found in adult MB. **c**, **e**, **f**, and **g** are images from *c309,eGFP*/ + ;*LpR1*^*DF*^/ + brains. **d** is an example from *c309,eGFP*/ + ;*LpR2*^*DF*^/ + brain; in green is shown GFP expression in MB neurons (*c309,eGFP*), in magenta is presented FasII staining to visualize the MB structure. Except **f**, all microphotographs correspond to a limited number of optical sections that help observe the phenotypes in each image. Empty arrowheads point to normal structure in MB lobes; full arrowheads show alterations. Scale bar: 20 µm. **h** Percentage of fly brains from each genotype exhibiting identified phenotypes; data is presented as strain (number of brains, number of independent experiments): *c309,eGFP* control animals (40, 4); *c309,eGFP*/ + ;*LpR1*^*DF*^/ + (41, 4); *c309,eGFP*/ + ;*LpR1*^*DF*^*/*LpR1^DF^ (35, 2); *c309,eGFP*/ + ;*LpR2*^*DF*^/ + (46, 4); *c309,eGFP*/ + ;*LpR2*^*DF*^/*LpR2*^*DF*^ (31, 2). Fisher test *P* = 6.46 × 10^−9^. **i** Distance between β lobes in each side of the fly brain in the different genotypes. Data expressed as mean ± SEM from the number of brains identified in **h**; one-way ANOVA, Kruskal Wallis post-test, *p* = 0.1486, “ns,” not significant. **j** Percentage of brains exhibiting phenotypes in flies expressing RNAi for LpR1 or LpR2 in MB neurons. Fisher test *P* = 1.02 × 10^−13^. **k** Distance between β lobes in knockdown strains. Data in **j** and **k** from *c309,eGFP* control flies (26, 4); RNAi-*LpR1*/ + control flies (15, 1); *c309,eGFP*/ + ;RNAi-*LpR1*/ + (21, 4); RNAi-*LpR2*/ + control flies (18, 1); *c309,eGFP*/ + ;RNAi-*LpR2*/ + (27, 4). One-way ANOVA, followed by Kruskal Wallis test *p* < 0.0001; * and ****, *p* = 0.0206 and *p* < 0.0001 respectively; “ns,” not significant

The principal neurons in the MB, the Kenyon cells, organize their dendrites and axons to give rise to the Calyx, peduncles, and lobes (Fig. 2a) [43, 44]. The Calyx, localized in the posterior aspect of the fly brain, is a structure formed by the Kenyon cells soma and dendrites, while their axons project frontally, arranged in a bundle called the peduncle. Before the end, the axonal fibers turn perpendicularly to give rise to the so-called MB lobes. The lobes are classified in γ, α/β, and α’/β’, where β, β’, and γ lobes are positioned horizontally while α and α’ lobes are placed vertically [45]. This anatomical organization has functional roles in several behaviors [46–48], so we paid close attention to MB organization in our studies.

We evaluated fly brains from adult animals deficient in LpR1 or LpR2 expression. Our strategy comprised several different genetic tools for each gene: a deletion mutant for each LpR (described previously in the functional experiments), an insertion mutant for each LpR, and RNAi against the receptors’ transcripts specifically expressed in the MB using the Gal4-UAS system.

In the analysis of results obtained in mutants for LpR1 or LpR2, we found and classified a variety of phenotypes (Fig. 2c–g) as compared to control brains (Fig. 2b). These include mislocalized lobes (Fig. 2c) in which the positions of one or more lobes are altered; the classification includes guidance defects where the α or β axons are misguided along β or α axons, respectively, resulting in thicker lobes. In some cases, we observed that the distance between MB β lobes is reduced (Fig. 2d), while in other brains, we detected fascicles of axons from indistinguishable origin crossing the middle line, which results in partial or total β lobes fusion (Fig. 2e). Further, we detected brains in which a part of the structure of the MB was lost; for instance, the absence of the lobes (Fig. 2f), the presence of thinner or shorter lobes (Fig. 2g), and very rarely, the complete lack of the MB (data not shown). These phenotypes were classified, as shown in Fig. 2h,i and in the Additional file 2: Table S1.

In general, flies lacking one copy of the *LpR1* gene (*c309,eGFP*/ + ; *LpR1*^*DF*^/ +) showed a higher percentage (17%) of brains exhibiting any alteration in MB than flies lacking one copy of *LpR2* (*c309,eGFP*/ + ;*LpR2*^*DF*^/ +): (6.5%) (Fig. 2h). These differences are exacerbated in knockout animals for each of the LpRs (Fig. 2h). Thus, the knockout animals for LpR1 (*c309,eGFP*/ + ; *LpR1*^*DF*^/*LpR1*^*DF*^) display a 22.9% of brains with MB alterations, while in the knockouts for LpR2 (*c309,eGFP*/ + ; *LpR2*^*DF*^/*LpR2*^*DF*^) a 25.8% of the brains exhibit any anatomical alteration. The analysis of the distance between β lobes indicated that this defect is not present in mutants for LpR1 and LpR2 (Fig. 2i).

To corroborate these results, we evaluated the MB architecture in insertion mutant animals (Additional file 1: Fig. S4a, Additional file 2: Table S2) where we found similar results: 22.8% of the haploinsufficient animals for LpR1 (*LpR1 CRIMIC*/ +) show alterations in the MB structure, while in the homozygous mutant flies (*LpR1 CRIMIC*/*LpR1 CRIMIC*) this percentage increased to 53.3% (Additional file 1: Fig. S4a). Additionally, the distance between β lobes was reduced in the LpR1 mutant flies (Additional file 1: Fig. S4b). Regarding LpR2, the heterozygous mutant animals (*LpR2 CRIMIC*/ +) exhibit 18.9% of brains with MB alterations, while in the homozygous mutant flies (*LpR2 CRIMIC*/*LpR2 CRIMIC*) it is possible to record 26% of brains with MB alterations (Additional file 1: Fig. S4a).

We attempted two additional approaches to assess the importance of LpRs in MB phenotypes. First, we studied whether it was possible to rescue the MB phenotypes observed in LpR2 mutants, by doing a genomic rescue. For this, we generated LpR2 mutant flies that also contain a genomic segment where the wildtype LpR2 gene is included (*LpR2 20 Kb*,BAC/ + ;*LpR2 CRIMIC*/ + and *c309,eGFP*/*LpR2 20 Kb*,BAC;*LpR2*^*DF*^/ +). The animals with the genomic rescue exhibited a normal MB architecture (Additional file 1: Fig. S4a).

To further address the importance of LpR1 and LpR2 specifically expressed in the MB, we evaluated potential defects in this structure in adult flies expressing RNAi under the control of the c309 driver (Additional file 1: Fig. 2j, k and Additional file 2: Table S3). Similar to data obtained with LpR mutants, the percentage of brains with MB alterations was higher in animal LpR1 knockdown (around 48%) as compared with LpR2 knockdowns (around 26%) (Additional file 1: Fig. 2j). On the other hand, the distance between MB β lobes was found significantly diminished only in animals expressing the RNAi for LpR1 (Fig. 2k).

To confirm that these defects were triggered by the reduced expression of LpRs and not by an artifact of the MB driver used (*c309*-Gal4), we expressed the RNAi for the LpRs under the control of a second driver: *OK107*-Gal4 [49] (Additional file 1: Fig. S4 and Additional file 2: Table S4). This driver is strongly expressed in the whole MB but also exhibits significant expression in some neurons that do not belong to the MB [43]. The structure of MB was similarly affected in animals expressing RNAi for LpR1 or LpR2 (phenotypes were observed in 50% and 41.4% of the brains analyzed, respectively) (Additional file 1: Fig. S4d). It was evident that the OK107 driver was associated with more severe phenotypes, including the disappearance of entire lobes. Similar to what was found using the c309 driver, the distance between β lobes was reduced in the MB after LpR1 knockdown (Additional file 1: Fig. S4d).

Overall, these results suggest that LpR1 and LpR2 play a relevant role in the proper development and establishment of the adult MB structure.

### Lipophorin receptor expression in mushroom body at distinct developmental stages

Our results support the idea that LpRs contribute to the establishment of the typical anatomical organization of adult *Drosophila* MB so that flies deficient in these receptors exhibit alterations in this brain structure. To advance on this idea, we decided to study the expression of LpR1 and LpR2 in MB at distinct developmental stages in *Drosophila*. For performing this task, we used some of the tools described above.

**Fig. 3:**
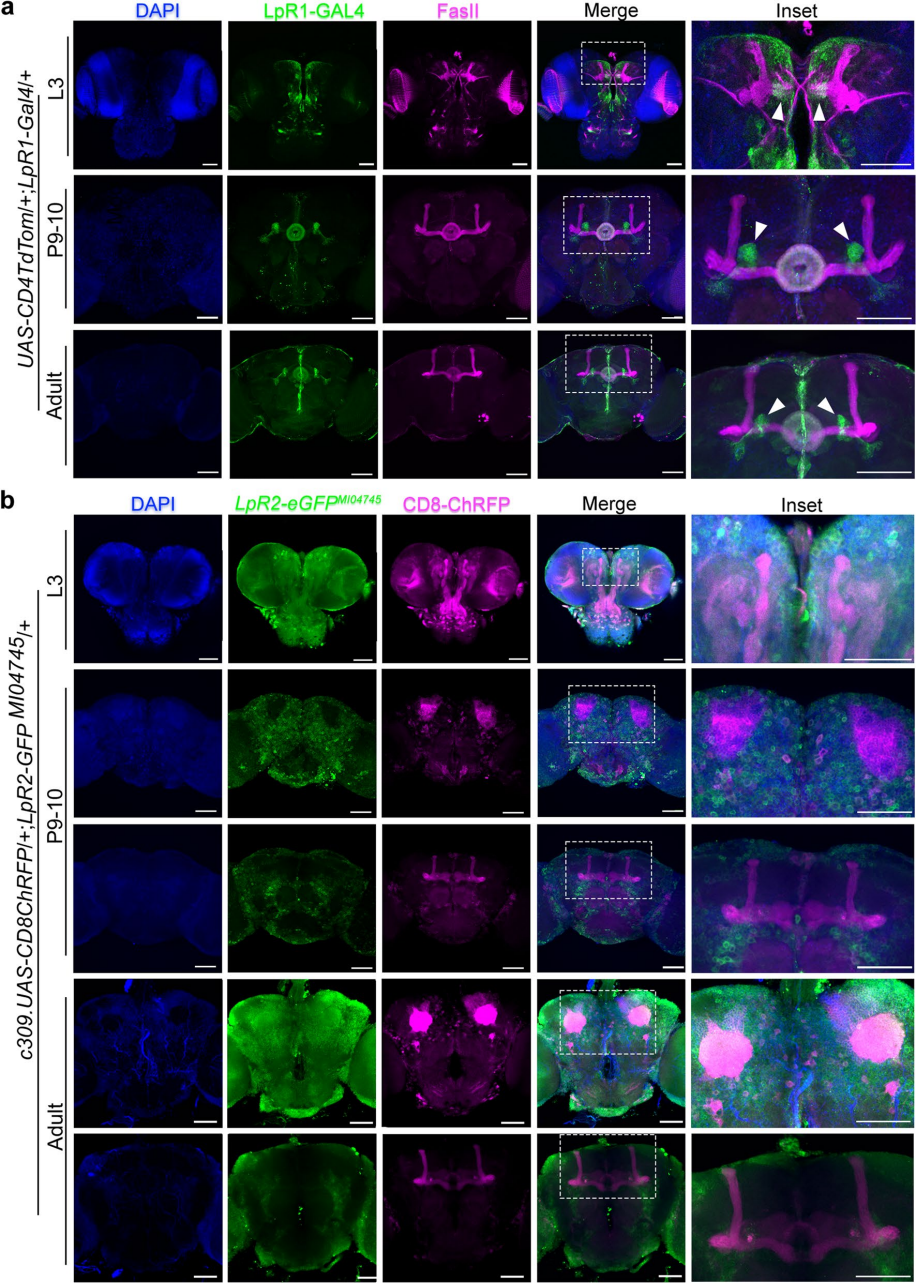
LpR expression in the brain throughout development. **a** Representative image of a fly brain that expresses *CD4::tdTomato* (green in the image) under the LpR1-Gal4 driver in third instar larvae (out of 11 brains), pupae stage 9–10 (2 brains), and adult animals 1–5 days old (10 brains). Immunofluorescence against FasII is shown in magenta and DAPI staining in blue. A discontinuous white line rectangle indicates the zone magnified in the insets. Scale white bars represent 50 µm. **b** Representative image of brains from third instar larvae (out of 16 brains), pupae stage 9–10 (15 brains), and male 1–5-day-old adult (13 brains) *LpR2-GFP*^*MI04745*^ animals, where LpR2 expression is followed by GFP (green in the image) and CD8-ChRFP expression in the MB is under the control c309 driver (magenta in the image). DAPI staining is in blue. Discontinuous white line rectangles indicate the zone magnified in the insets. Scale white bars represent 50 µm

By using the Gal4-UAS system to command the expression of fluorescent proteins under the control of an LpR1 driver, we found that in the adult brain LpR1 was highly enriched in a group of neurons found adjacent to the MB β/β’ lobes and in the central complex (Fig. 3a, inset, arrowheads), while its expression in the rest of the brain was rather diffuse. The cells showing the highest LpR1 expression in the fly brain seem to be the neurons projecting to the ring of the ellipsoid body [50–52]. As an alternative approach, we used a LpR1 antibody previously reported [13] and FasII staining to identify the MB (Additional file 1: Fig S5). Both strategies provided similar results. We also assessed the expression of LpR2 in the adult fly brain, employing a protein trap line generated by Recombination-Mediated Cassette Exchange (RMCE). In this line, the RMCE cassette is placed between the exons 12 and 13 of LpR2 and contains a GFP coding-exon that tags all the LpR2 isoforms [53]. Our data showed that LpR2 was not enriched in any specific brain region (Fig. 3b). These results suggest that both LpR1 and LpR2 exhibit a rather diffuse expression throughout the adult fly brain, although LpR1 is highly expressed in a group of cells surrounding the MB.

Considering the evidence obtained in adult flies, we analyzed the expression of LpRs in MB over larval and pupal stages, posing the idea that the alterations in adult MB structure could be explained by a modification in the developmental program that gives rise to this fly brain region. The MB begins to develop in the embryonic stage, starting from four neuroblasts per hemisphere that divide until the late pupa stage. The γ lobe develops until the mid-third instar larval stage. The α’ and β’ lobes are generated by the end of the third larval stage, while the development of α and β lobes reach maturity at the pupal stage [54, 55].

Using the LpR1 Gal4 tool, we assessed the expression of LpR1 in the larval and pupal stages. At the larval stage, LpR1 was found at low levels in the horizontal γ lobe, with a widespread expression in the midline area of the brain and the photoreceptors, while in pupae, the receptor was found highly expressed in the central complex and in a group of neurons that surround the horizontal lobes (Fig. 3a, arrowheads), similarly to what was found in adult flies. On the other hand, the RMCE tool was used to assess the expression of LpR2 in the larval and pupal stages. In larvae, LpR2 was observed in cells located in the midline area and the photoreceptors, whereas in pupae, it was observed in cells in the brain cortex (Fig. 3b). These results indicate that LpR1 and LpR2 expression is widely distributed in the fly brain cells throughout development, and suggest that they play a relevant role in the proper development and establishment of the adult brain MB structure.

### LpRs contribute to Drosophila MB neurite development

**Fig. 4:**
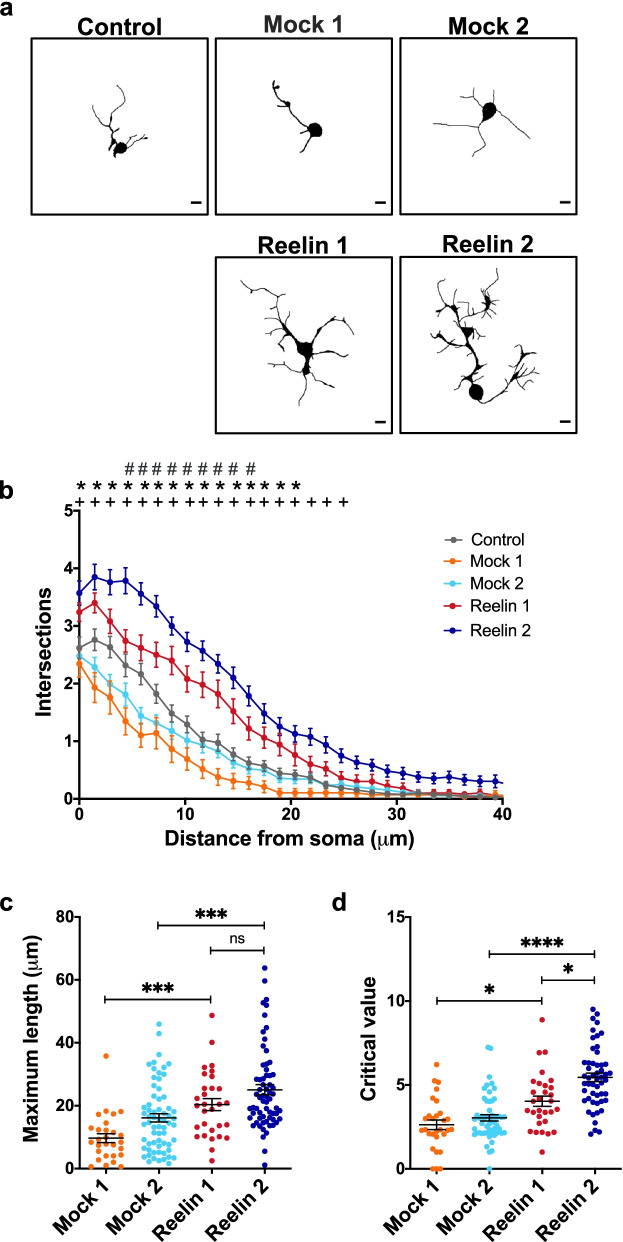
Mushroom body neurons respond to Reelin. **a** Representative images of MB neurons in primary cultures of the *c309,eGFP* strain, after Mock or Reelin treatment. 13 nM Reelin and 30 nM Reelin treatment correspond, respectively, to 20 and 50% dilution of Reelin conditioned medium in the cell culture. Mock 1 and Mock 2 treatments are the experimental controls of 13 and 30 nM Reelin, respectively. MB neurons are identified by GFP expression. Black bars in the lower right corner represent 5 µm. **b** Sholl profile of MB neurons after different treatments. Data are expressed as mean ± SEM; two-way ANOVA, *p* < 0.0001 for each factor. “*” indicates a significant difference (*p* < 0.05) between the 13 nM Reelin and the corresponding Mock 1 treatment, at a given distance from the soma; “ + ”, significant difference (*p* < 0.05) between 30 nM Reelin and Mock 2 treatment, at the same distance from the soma. “#”, (*p* < 0.05) between effects at 13 and 30 nM Reelin concentrations at the same distance from the soma. **c** Maximum length, defined as the greater distance reached for a cell’s neurite with respect to its soma, recorded in MB neurons under the different treatments. Data expressed as mean ± SEM; one-way ANOVA, followed by Kruskal Wallis post hoc test, *p* < 0.0001. *** indicates *p* < 0.0005. **d** Critical value, the number of neurites found in the radius with most neurites, in MB neurons under each treatment. Data is mean ± SEM; one-way ANOVA and Kruskal Wallis post-test, *p* < 0.0001. *, ****, are *p* < 0.05, *p* < 0.0001. “ns” not significant. Data from *n* = 20 coverslips in four independent experiments, 15–20 cells in each “*n*”

The proposed orthologs for LpR1 and LpR2 in vertebrates are ApoER2 and VLDL-R [2, 6]. These membrane proteins are the primary receptors for Reelin in CNS [23, 29, 56] and participate in the development and function of the cerebral cortex and hippocampus, among other brain areas. Besides LpRs, *Drosophila* has orthologs for other proteins involved in Reelin signaling, including the adaptor protein Dab (see below). Thus, we proposed that *Drosophila* neurons in culture would respond to mammalian Reelin, increasing their neurite complexity, as reported in cultured rodent neurons [57–60]. Strikingly, we found that our prediction was correct: MB neurons significantly increased their neurite branching in response to mammalian Reelin compared to effects induced by mock media (Fig. 4). Moreover, the response changed depending on the concentration of Reelin used (Fig. 4a, b); the maximum effect was observed at a concentration of 30 nM similar to that previously used in mammalian hippocampal and cortical neurons [60, 61]. Several parameters highlighting the complexity of the neurite arborization were studied. For instance, Reelin treatment increased the maximum length that neurites can reach compared with mock-treated neurons (Fig. 4c). Besides, Reelin exposure also increased the distance (from the soma) in which the maximum number of neurites is observed (critical value) (Fig. 4d).

**Fig. 5:**
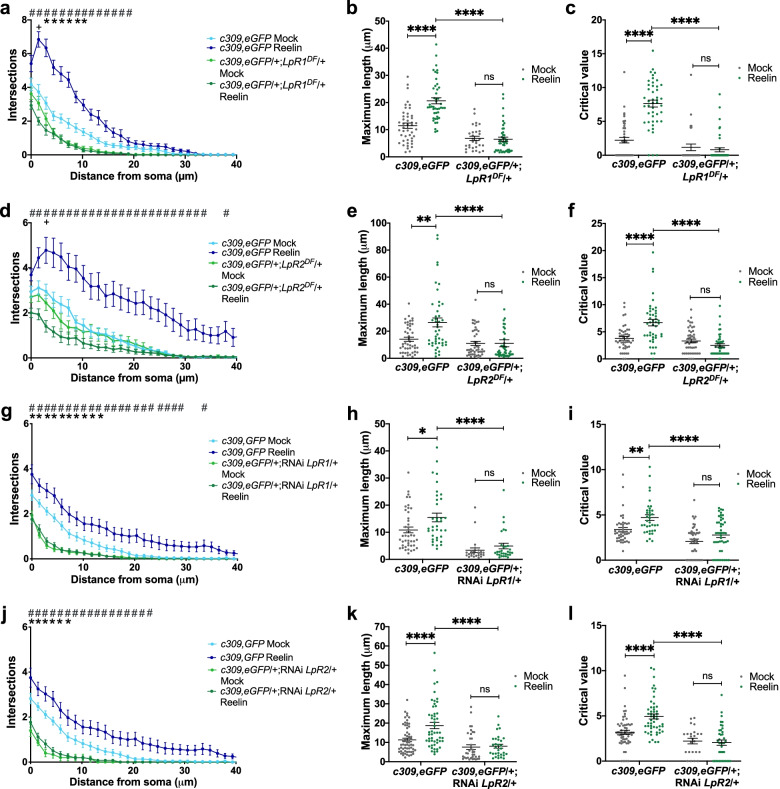
Reelin effects on mushroom body neurons depend on LpR1 and LpR2.** a**–**f** Reelin does not affect neurite complexity in mutants for LpRs. **a, d** Sholl profile of cultured MB neurons from LpR1 (*c309,eGFP*/ + ;*LpR1*^*DF*^/ +) or LpR2 (*c309,eGFP*/ + ;*LpR2*^*DF*^/ +) mutant flies and their controls (*c309,eGFP*), after Mock or Reelin treatment (30 nM). Two-way ANOVA shows that treatment and genotype factors and their interaction play a role in results obtained for each data set (*p* < 0.0001 for all). “#” shows significant differences (*p* < 0.05) between the genetic control and mutants after Reelin treatment. “*” indicates significant differences between mutant neurons and genetic control after Mock treatment. “ + ” means a significant difference (*p* < 0.05) between mock and Reelin treatment in the mutant genotype. **b, e** Maximum length. **c, f** Critical value in the genotypes identified, after mock or Reelin treatment. In **b**, **c**, **e,** and **f,** two-way ANOVA shows that treatment and genotype factors, and also the interaction between factors, play a role in results (*p* < 0.0001 for each analysis); ** and ****, indicates *p* < 0.01 and *p* < 0.0001 between conditions. Data in **a**–**f**, from *n* = 24 coverslips, 15 cells studied in each coverslip, three independent experiments. **g**–**l** Reelin does not affect neurite complexity in LpRs knockdown flies. **g, j** Sholl profile, **h, k** maximum length, and **i, l** critical value measured in flies expressing RNAi for either LpR1 or LpR2 in MB neurons (*c309,eGFP*/ + ;RNAi-*LpR1*/ + or *c309,eGFP*/ + ;RNAi-*LpR2*/ + , respectively), as compared to controls (*c309,eGFP*), after Mock or Reelin treatment. In **g, j,** two-way ANOVA shows that treatment and genotype factors and their interaction play a role in results obtained for each data set (*p* < 0.0001 each). “#”, significant differences (*p* < 0.05) between control and LpRs knockdowns after Reelin treatment. “*”, significant differences between knockdown and control after Mock treatment. No significant differences were observed between mock and Reelin treatment in the knockdowns for LpRs. In **h**, two-way ANOVA indicates that only genotype and treatment factors play a role in results (*p* < 0.0001 and *p* = 0.0170 respectively). In **k**, two-way ANOVA indicates that genotype and treatment factors and their interaction play a role in results (*p* < 0.0001, *p* = 0.0034, and *p* = 0.0096 respectively). In **i**, two-way ANOVA indicates that only genotype and treatment factors play a role in results (*p* < 0.0001 and *p* = 0.0002 respectively). In **l**, two-way ANOVA indicates that genotype and treatment factors and their interaction play a role in results (*p* < 0.0001, *p* = 0.0073, and *p* = 0.0012 respectively). *, **, and **** indicated *p* < 0.05, *p* < 0.01, and *p* < 0.0001 respectively. Data in **g**–**l**, from *n* = 12 coverslips, 10–15 cells studied in each coverslip, three independent experiments. Data in **j**–**l**, *n* = 16 coverslips, 10–15 cells studied in each coverslip, four independent experiments. ns, not significant

Next, we determined the role of LpRs in the morphological effects induced by Reelin in primary culture neurons. First, we verified the expression of LpRs in *Drosophila* brain cultured neurons (Additional file 1: Fig. S6). Then, LpR1- or LpR2-deficient flies were used to prepare neuronal cultures that were exposed to 30 nM of Reelin. Our results showed that the LpR-deficient MB neurons did not respond to Reelin, while responses to this signaling molecule were evident in cultured MB neurons from control animals (Fig. 5). Interestingly, under basal conditions, LpR1 mutant neurons already showed impaired neurite development (Fig. 5a). Other defects were also evidenced when studying the maximum length of neurites (Fig. 5b) and the critical value (Fig. 5c). Similarly, neuronal cultures derived from LpR2 mutant animals did not respond to Reelin, although a basal alteration in neurite development was not observed (Fig. 5d–f).

To confirm these results and dissect the contribution of LpRs specifically in MB neurons, we took advantage of flies expressing RNAi for LpR1 or LpR2 only in the MB by using the Gal4-UAS system. By performing the same type of experiments outlined above, we obtained similar results to those described in cultured neurons from mutant flies: the inability of MB neurons to respond to Reelin when expressing RNAi for LpRs (Fig. 5g–l). Overall, these results show for the first time that vertebrate Reelin stimulates the growth and neurite complexity of *Drosophila* MB neurons in a way that depends on the expression of LpRs.

### LpRs long isoforms are required to mediate Reelin internalization

The interaction of Reelin with ApoER2 and VLDL-R requires the LA modules in the extracellular domain of the receptors [21, 24], which are conserved in LpRs [2, 13]. Since the main difference between the different isoforms for LpRs is the presence of the first LA module plus the non-conserved domain, we asked whether the effect observed in neuronal cultures exposed to vertebrate Reelin depends on specific isoforms of LpRs. In order to work on this question, we used the insect cellular model S2, which does not express LpRs [16].

**Fig. 6:**
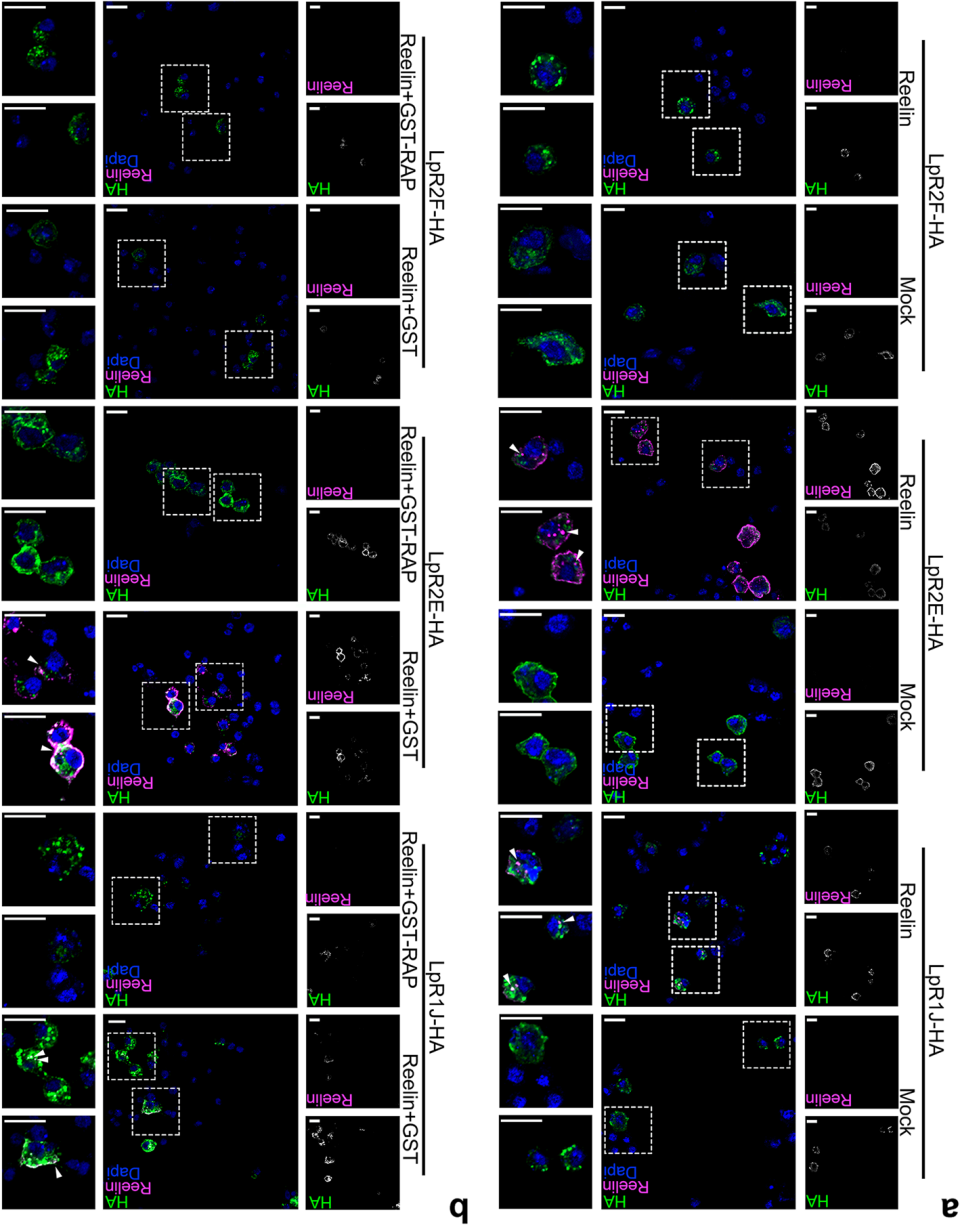
LpRs long isoforms internalize Reelin.** a** Representative images of S2 cells transfected with either LpR1J-HA, LpR2E-HA, or LpR2F-HA and treated with 30 nM Reelin or the equivalent volume of Mock media. Results of immunofluorescence against HA and Reelin. The panels in the right show magnifications of the area presented in the discontinuous white line square in the center image; data obtained from *n* = 2 coverslips in one experiment. The lower right white bar in images represents 10 µm. **b** Representative images of S2 cells transfected either LpR1J-HA, LpR2E-HA, or LpR2F-HA and treated with 30 nM Reelin plus 500 nM GST-RAP or 30 nM Reelin plus 500 nM GST. Results of immunofluorescence against HA and Reelin. The panels in the right show magnifications of the areas within the discontinuous white line square in the center image; data obtained from *n* = 2 coverslips in one experiment. The lower right white bar represents 10 µm

To test a functional Reelin-LpR interaction, we carried out a ligand internalization assay in which S2 cells were transiently transfected with the following available tools: two long isoforms of LpRs tagged with an HA epitope (i.e., LpR1J-HA or LpR2E-HA) and one short isoform of LpR2: LpR2F-HA (Fig. 6a, Additional file 1: Fig. S7). Reelin was internalized only in cells expressing the longer forms of the receptors, either LpR1J-HA or LpR2E-HA (Fig. 6a). Meanwhile, S2 cells transfected with the LpR2F isoform (Fig. 6a) or the empty plasmid (Additional file 1: Fig. S8a) did not internalize Reelin.

The Receptor-Associated Protein (RAP) is a chaperone required for folding the LDL-R family members. RAP binds with high affinity to the ligand-binding domains of the receptors, preventing their premature interaction with ligands within the endoplasmic reticulum [62]. Several antecedents show that the extracellular addition of recombinant RAP prevents the binding of LDL-R family members to their respective ligands, including the binding of VLDL-R and ApoER2 with Reelin [35, 62–64]. In addition, Van Hoof et al. [65] showed that the internalization of *Locusta migratoria* Lipophorin by its LpR is completely inhibited by adding the recombinant form of the human RAP. Then, to evaluate whether Reelin internalization depends on a classical interaction with lipoprotein receptors, the internalization experiment was performed in the presence of RAP (Fig. 6b, Additional file 1: Fig. S8b). Reelin entrance to the cells, mediated by LpR1J or LpR2E, was prevented by the presence of RAP in the media (Fig. 6b).

Overall, these data suggest an interaction between mammalian Reelin and the two longer LpRs isoforms, which could underlie a novel, previously not described, signaling cascade.

### Dab is required for Reelin-induced responses in MB neurons

**Fig. 7:**
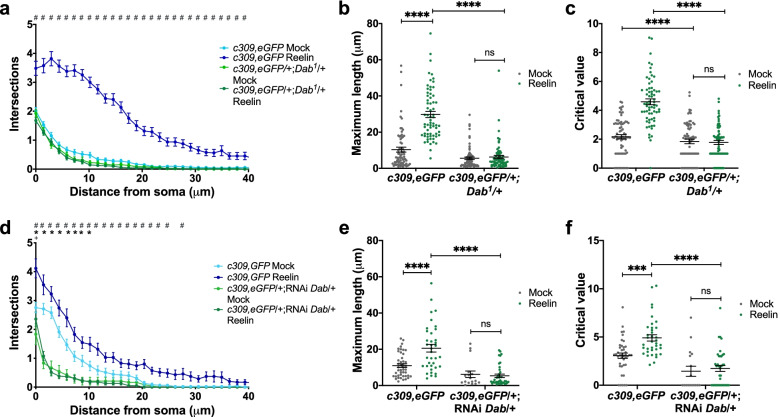
Reelin effects on mushroom body neurons depend on Dab. **a**–**c** Reelin does not increase neurite complexity in neurons from Dab mutant flies. **a** Sholl profile of cultured MB neurons from Dab (*c309,eGFP*/ + ;*Dab*^*1*^/ +) mutant flies and their controls (*c309,eGFP*), after Mock or Reelin treatment. Two-way ANOVA shows that treatment and genotype factors and their interaction play a role in results obtained (*p* < 0.0001 for all). “#”, significant differences (*p* < 0.05) after Reelin treatment between the genetic control and all other experimental groups. There were no significant differences between all other conditions. **b** Maximum length and **c** critical value in mutant and control flies, after mock or Reelin treatment. In **b, c,** two-way ANOVA shows that treatment and genotype factors and the interaction between factors play a role in the results (*p* < 0.0001 for each analysis); ****, indicates *p* < 0.0001 between conditions. **d**–**f** Reelin does not affect neurite complexity in knockdown animals for Dab. **d** Sholl profile, **e** maximum length, and **f** critical value measured in flies expressing an RNAi for Dab only in MB neurons (*c309,eGFP*/ + ;RNAi-*Dab*/ +), as compared to controls (*c309,eGFP*), after Mock or Reelin treatment. In **d**, two-way ANOVA shows that treatment and genotype factors and their interaction play a role in results obtained for each data set (*p* < 0.0001 for all). “#”, significant differences (*p* < 0.05) between control and Dab knockdown after Reelin treatment. “*”, significant differences between knockdown and control, after Mock treatment. “ + ”, significant differences (*p* < 0.05) between mock and Reelin treatment on the knockdown fly strain. In **e, f**, two-way ANOVA indicates that genotype and treatment factors, and their interaction play a role in results (*p* < 0.0001, *p* < 0.01, and *p* < 0.05 respectively for each condition). *** and ****, *p* < 0.005 and *p* < 0.0001; “ns”, not significant. Data in **a**–**f**, from *n* = 12 coverslips 4–20 cells studied in each coverslip, three independent experiments

Reelin signaling requires the dimerization of ApoER2 or VLDL-R and the concomitant recruitment of the cytosolic adaptor protein Dab1 to the NPxY motif present in the cytoplasmic tail of these receptors [30, 35, 66]. In *Drosophila*, Disabled (Dab) is the homologous form of vertebrate Dab1 [33, 67]. To determine whether Dab contributes to the Reelin-induced response in MB neurons in culture, we used a mutant fly lacking one copy of *Dab* [68]. In our hands, homozygous mutants for this gene die before reaching the third instar larvae. The reduction of Dab expression in this mutant was corroborated by qPCR (Additional file 1: Fig. S9a). We carried out the same type of in vitro experiments described when assessing the role of LpRs in primary neurons, but now in cultures prepared from *Dab* heterozygous mutants (Fig. 7a–c). The Dab mutant neurons failed to respond to Reelin treatment: there was no change in neuronal arborization (Fig. 7a), maximum length (Fig. 7b), or the critical value (Fig. 7c). As a complementary approach to corroborate these findings, Reelin was added to cultures prepared from brains expressing RNAi for Dab specifically in the MB, using c309-Gal4 (Fig. 7d–f). The diminished expression of Dab was verified through qPCR (Additional file 1: Fig. S9b). As with the Dab mutant cultures, Reelin treatment did not increase the complexity of neurite branching (Fig. 7d), the maximum length (Fig. 7e), or the critical value (Fig. 7f) in MB neurons expressing RNAi for Dab. These results show that Dab plays a relevant role in the response of MB neurons to Reelin.

### Dab is required for MB development and function

**Fig. 8:**
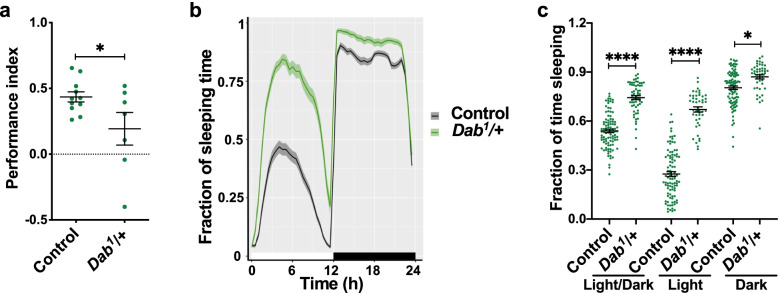
Dab is required for olfactory memory and establishing MB structure.** a** Memory performance is reduced in *Dab*^*1*^ mutants as compared to control animals. Data is mean ± SEM; *n* = 7 independent experiments, *t*-test, *p* = 0.0407 (* significant difference). **b** Sleep profile in *Dab* mutant flies. The white bar above the *x*-axis represents the hours of the day when flies were exposed to light; the black bar represents hours when flies were exposed to darkness. **c** Fraction of sleeping time in *Dab* mutants. Data in **b** and **c**, from *n* = 2 independent experiments, 15–25 flies studied per genotype in each *n*. Data is mean ± SEM. Two-way ANOVA shows that light and genotype factors, and also the interaction between factors, contribute to results (*p* < 0.0001 for each analysis), Tukey post-test; *, and ****, indicates *p* < 0.05, and *p* < 0.0001 between conditions

To determine whether mutant flies for Dab exhibit alterations in olfactory memory, we carried out the training protocol as outlined for *LpR* mutants. The performance index of flies lacking one copy of Dab was significantly lower than that of control animals (Fig. 8a). These flies exhibited a normal response to the electric shocks and the odorants used (Additional file 1: Fig. S10 a-c). Additionally, we evaluated sleep patterns in animals deficient in Dab expression (Fig. 8b, c). Mutant flies exhibited higher sleep time throughout the day, regardless of the phase (Fig. 8b, c). The sleep patterns were also assessed in flies expressing an RNAi against Dab in the MB (Additional file 1: Fig. S11). Interestingly, flies knockdown for Dab in the MB exhibit a normal sleep pattern (Additional file 1: Fig. S11). Overall, these data suggest that Dab contributes to sleep homeostasis, but not by an action in the MB.

**Fig. 9:**
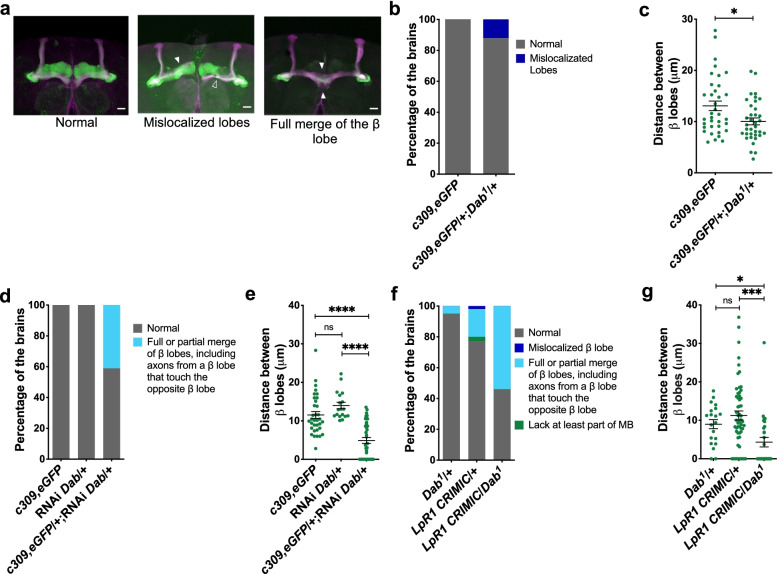
Dab interact with LpR1 for the correct establishment of the MB structure.** a** Representative images of the phenotypes observed in MB of flies lacking one copy of Dab (*c309,eGFP*/ + ;*Dab*^*1*^/ +) or that express RNAi against Dab in MB neurons (*c309,eGFP*/ + ; RNAi-*Dab*/ +). Green, GFP; Magenta, Fas II immunostaining. Empty arrowheads show normal MB structure; full arrowheads indicate structural alterations. White bar: 20 µm. **b** Distribution of phenotypes observed in control *c309,eGFP* (*n* = 36 brains, 4 experiments) and haploinsufficient mutant *c309,eGFP*/ + ;*Dab*^*1*^/ + (*n* = 34 brains, 4 experiments) animals. Fisher test, *p* = 3.44 × 10^−4^. **c** Distance between β lobes in brains from Dab haploinsufficient mutants as compared to controls; *t*-test, shows significant differences (*p = 0.0107); data in *c309,eGFP* from *n* = 36 brains, 4 experiments, and *c309,eGFP*/ + ;*Dab*^*1*^/ + from *n* = 40 brains, 4 experiments. **d** Relative percentage for phenotypes observed when RNAi against Dab transcript is expressed in MB neurons. Fisher test, *p* = 2.2 × 10^−16^ in *c309,eGFP* (*n* = 26 brains, 3 experiments,), *RNAi-Dab/* + (*n* = 18 brains, 1 experiments) and in *c309,eGFP/RNAi-Dab* (*n* = 34 brains, 3 experiments) flies. **e** Distance between β lobes in flies expressing RNAi for Dab in MB neurons as compared to control animals; one-way ANOVA (*p* < 0.0001) followed by Kruskal Wallis. ****, *p* < 0.0001. Data from *c309,eGFP* (*n* = 26 brains, 3 experiments), *RNAi-Dab/* + (*n* = 18 brains, 1 experiments) and in *c309,eGFP/RNAi-Dab* (*n* = 34 brains, 3 experiments). **f** Relative percentage of brains with MB defects in double LpR1 and Dab heterozygous mutants (*LpR1 CRIMIC/Dab*^*1*^*)* as compared with haploinsufficient animals for each gene. Data obtained from *Dab*^*1*^*/* + animals in *n* = 20 brains, 2 experiments*; LpR1 CRIMIC/* + animals in *n* = 57 brains, 4 experiments (same brains used in the Additional file 1: Fig. S4); and *LpR1 CRIMIC/Dab*^*1*^ in *n* = 28 brains, 2 experiments. Fisher test, *p* = 3.7 × 10^−16^. **g** Distance between β lobes in double *LpR1 CRIMIC/Dab*^*1*^ mutant animals as compared to heterozygous mutants in each gene. Data obtained from *Dab*^*1*^*/* + animals in *n* = 20 brains, 2 experiments*; LpR1 CRIMIC/* + animals in *n* = 57 brains, 4 experiments (same brains used in the Additional file 1: Fig. S4); and *LpR1 CRIMIC/Dab*^*1*^ in *n* = 28 brains, 2 experiments. One-way ANOVA (*p* = 0.0002) followed by Kruskal Wallis. * and ** means *p* = 0.0156 and *p* = 0.0002 respectively. “ns” means not statically significant

We also studied the contribution of Dab to MB organization by using two different genetic tools: mutants generated by a deletion in the Dab gene or animals expressing, only in MB neurons, an RNAi directed to transcripts of this gene (Fig. 9, Additional file 2: Table S5-S7). The phenotypes observed in the Dab-deficient animals included mislocalized lobes in 11.7% of brains (Fig. 9a, b). Besides, Dab mutants exhibit a lower distance between β lobes (Fig. 9c). On the other hand, 41.2% of the brains from flies expressing RNAi against Dab exhibit MB with β lobes fused or axons crossing from one β lobe to the opposite (Fig. 9a, d, e). Therefore, compared to controls, MB from Dab-deficient flies exhibit their β lobes closer.

Finally, to evaluate the possibility of a genetic interaction between Dab and LpRs, we assessed the MB architecture in animals deficient in one allele in Dab while in the other allele where deficient for LpR1 (Fig. 9f, Additional file 2: Table S7). In these new experiments, we recorded 5% of the brains with MB alterations in Dab haploinsufficient animals (*Dab*^*1*^/ +) (Fig. 9f). On the other hand, 22.8% of the LpR1 heterozygous mutants (*LpR1 CRIMIC*/ +) displayed anatomical phenotypes (Fig. 9f). Remarkably, flies bearing one copy of the mutant alleles for each gene (*LpR1 CRIMIC* / *Dab*^*1*^) exhibited 53% of the brains with alterations in the MB structure (Fig. 9f). In all of the brains, we observed merging of the MB lobes, and consistently, these flies presented β lobes very close to each other (Fig. 9g). These data are in support of the idea of a genetic interaction.

These results show that the presence of Dab is necessary for MB development and function in a similar way to LpRs.

## Discussion

Here we have demonstrated that LpR1, LpR2, and the adaptor protein Dab have a relevant and previously unnoticed role in the correct anatomical organization of adult MB and consequently in its associated functions, including olfactory memory performance and sleep patterns. Besides, the exposure of cultured *Drosophila* MB neurons to mammalian Reelin, a protein not present in flies, resulted in augmented neurite growth and complexity, an effect that depends on these receptors and Dab. Further, two of the LpRs long isoforms, LpR1J and LpR2E, are required for the internalization of Reelin in S2 cells.

### LpRs are required for adult MB structure and operation

The expression of LpR1 or LpR2 or the intracellular adapter Dab in MB is crucial for proper performance in memory assays. Despite we found a difference in the naive olfactory response recorded in LpR1 mutants as compared to control animals that constitute a confounding effect on olfactory memory results, the entire set of results support that there is an effect on olfactory memory, which is not surprising given the strong anatomical phenotypes observed in LpR1 mutants. Further experiments are required to evaluate whether LpRs expressed in specific MB subpopulations differentially contribute to olfactory memory. The MB, fly brain region linked to the generation and storage of olfactory memories [44, 69], is proposed to play a homologous function to the mammalian hippocampus [70–73]. The MB and the hippocampus categorize stimuli according to the type of signal received and the context in which the signals are received, fundamental aspects for learning and memory processes [71]. Moreover, numerous ortholog genes are required in the hippocampus and MB for similar functions [73].

On the other hand, LpRs also participate in normal sleep homeostasis, another function associated with MB [40, 41]. Whether LpRs are required in brain regions beyond MB for sleep homeostasis is an issue we did not explore in this work. Interestingly, Yin et al. [19] showed that the reshuffle of LNv dendrites under light stimuli was prevented when LpRs expression was eliminated [19]. Dab mutants also exhibited a higher sleep time throughout the day. Given the fact that sleep phenotypes were observed only with the mutants but not confirmed in animals expressing RNAi against Dab in MB, future studies should extend our analysis on how Dab influences sleep phenotypes. Further steps in assessing these issues could be also directed to evaluate how LpRs and Dab expressed in circadian regions contribute to sleep homeostasis.

The defects in MB structure observed in mutants for LpR1, LpR2, and Dab indicate the importance of these proteins in the appropriate organization of this fly brain region along development, determining its function in adult animals. Although we did not observe a high expression of LpR1 in the adult MB, our data show that it is enriched in the horizontal lobe in the third instar larvae, a stage where the MB cells are developing [48]. Our expression studies demonstrating diffuse expression of LpR1 and LpR2 in third instar larvae are coincident with previous reports [18, 19]. Interestingly, our results using the LpR1-Gal4 tool in adulthood and pupae stage showed that the receptor’s highest expression is not in the MB itself but in a group of cells surrounding the β’/β lobe of this structure. The complementary use of the LpR1 antibody supports the notion that these cells could be R neurons from the ellipsoid body [50–52]*.* Future experiments using ellipsoid body Gal4-specific drivers would be needed to demonstrate this idea. In addition, these data would suggest that MB extrinsic neurons, which exhibit the highest expression for LpR1 in fly brain, are somehow signaling to the MB neurons during development to generate the typical organization of adult MB. This proposal would also need evaluation. Remarkably, similar mechanism has been demonstrated for other proteins. For instance, Ptpmeg is a cytoplasmic tyrosine phosphatase highly expressed in the ellipsoid body and little expressed in adult MB. The reduction of Ptpmeg expression results in MB phenotypes, similar to those described here [74]. A similar situation occurs with the long-chain acyl-CoA synthetase 4 (ACSL4), a protein expressed at low levels in the MB but required for its adequate development [75]. Axonal patterning defects could underlie some of the phenotypes observed in animals deficient for LpRs [76]. Future studies on how MB structure is formed over development in LpR- and Dab-deficient animals could support these ideas.

Axonal patterning defects could underlie some phenotypes observed in animals deficient for LpRs [76]. For example, the fusion of the β lobes is an indication of excessive and uncontrolled growth of axons crossing toward the contralateral hemisphere. It could be speculated that axons bearing LpRs and Dab should sense a stop signal close to the midline. When some intrinsic or extrinsic components are not present, the β axons cross the midline. In this regard, it is worth highlighting a parallel with the role of ApoER2, VLDL-R, Dab1, and Reelin in the process of cell migration and axonal growth and targeting [77]. Reelin functions as an attractive cue for neuronal migration in the hippocampus and the cortex [23, 26, 78], as well as for axonal targeting in the visual system [79, 80] and the entorhinal-hippocampus circuit [81, 82]. Besides, Reelin behaves as a stop signal in the vicinity of cells secreting it (i.e., Cajal-Retzius in the developmental cortex and hippocampus). Therefore, in the absence of the receptors, Dab1 or Reelin, neurons invade the marginal zone [30, 67]. In the case of Reelin, the stop signal relies on the activation of Rho GTPases and LIMK [29, 83]. In *Drosophila*, the growth of MB axons of the α/β lobes is also controlled by attractive and repulsive cues, and in some cases, as for the TGF-β 1 receptor, the stop signal includes LIMK activation [84].

Other phenotypes observed upon LpRs knockdown are stalling of MB lobes. Previous studies show that overexpression of LIMK and some manipulation of Rho GTPases activity lead to MB axon stalling [85, 86], which is consistent with the role of Reelin in the control of these cytoskeleton regulators. Finally, defects in guidance in which the α and β lobes follow the same trajectory have been observed in mutants of the Eph-Ephrin [87] and Wnt5/Drl1/2 [88] signaling pathways, indicating LpR could be part of a complex signaling network regulating MB axon guidance.

Consistent with the structural and functional studies in the *Drosophila* brain, the experiments carried out in primary cultured neurons support the importance of LpRs in the structure of MB cells. Our data show impairment in basal neurite development in cultured neurons from mutants for these genes. The primary neuronal culture is a cell system generated from pupal brains, and therefore, phenotypes observed argue in favor of the idea that LpRs play a role in MB development.

Although the correlation between alterations in MB structure and behavioral phenotypes has been previously reported for several mutants of *Drosophila* genes [47, 89], to our understanding, this is the first time that this is shown for mutants in LpRs or the adaptor Dab. Overall, our results suggest that LpR and Dab regulate several aspects of MB morphogenesis and function, and future studies should address its interaction with other pathways involved in these processes.

### Hints on a novel function for LpRs

Regarding the function of LpRs in *Drosophila*, both receptors have been primarily associated with lipid uptake [12, 13, 16, 17]. Thus, disturbing LpR1 or LpR2 expression in early developmental stages reduces neutral lipid content in oocytes and imaginal discs. This phenotype is rescued by expressing long isoforms of LpRs, LpR1H, and LpR2E [13]. ApoD and ApoE are endocytosed in mammalian cells by ApoER2 and VLDL-R [1, 90]. Recent work from Yin et al. [18] shows that a short isoform of LpR1 (LpR1G) interacts with the lipocalin Glaz. Interestingly, a report from the Bellen group shows that the transport of lipids between neurons and glia is mediated by Glaz and neural lazarillo proteins (Nlaz), the orthologs for ApoD in flies [91]. The mechanism associated with lipid uptake by the glia is unknown. It is then possible to hypothesize that some of the alterations we described in MB in LpRs-deficient animals could be due to deficits in the transport of lipids between neurons and glia.

Interestingly, Matsuo et al. [16] showed that the lack of LpR1 and LpR2 does not significantly affect triglycerides levels in the CNS of third instar larvae, while the brain content of only few phospholipids seem to be affected in these deficient animals. This phenotype was also described by Yin et al. [18]. Since the highest expression for LpRs is described at the larval stage, the work of Matsuo et al. has several implications. First of all, it indicates that there are probably other complementary mechanisms responsible for the uptake of neutral lipids, so that deficiency on LpRs is not associated with a dramatic decrease in larval lipid content. Also, it supports the idea that LpRs could play other roles in addition to lipid uptake. In this regard, it was shown that LpR1 and LpR2 are required for proper LNv dendrite development and that these receptors are necessary for LNv structural plasticity in response to light stimuli in third instar larvae [19]. The mechanisms by which the light-induced increase in synaptic activity is related to LpR function were not discussed [19].

Strikingly, our data show that cultured MB neurons respond to Reelin treatment in a way depending on the expression of LpRs and Dab. The Reelin-induced increase in neurite arborization is similar to what has been previously described in cultured hippocampal neurons [57–60]. Likewise, the absence of a Reelin-induced response in cultured fly neurons deficient in LpRs is similar to the situation observed in cultured vertebrate neurons when the function of ApoER2 and VLDL-R is inhibited [59, 61].

Our results also show that the long isoforms of LpR1 and LpR2, LpR1J and LpR2E, respectively, bind and internalize mammalian Reelin in *Drosophila* cells. Since we only evaluated some of the LpR isoforms, we cannot discard that other LpRs would mediate Reelin interaction and internalization or that other long isoforms participate in MB development. Nevertheless, our results suggest that LpRs short isoforms would have a different role than the two long isoforms.

The prevention of Reelin internalization by RAP indicates that Reelin would bind LpR1J and LpR2E isoforms through its cysteine-rich repeats in the LA module, like the rest of the LDL-R family members [6, 55, 83]. Reelin signaling also requires the interaction of Dab1 through its PTB domain with the NPxY motif of ApoER2 and VLDL-R [33, 35, 92]. As mentioned, the same motif is found in LpR1 and LpR2 [13], while the PTB domain is conserved in Dab from *Drosophila melanogaster* [33]. Furthermore, once Dab1 binds to ApoER2 or VLDL-R, it is phosphorylated in two groups of tyrosine residues [66], and it is already known that *Drosophila* Dab tyrosines can be phosphorylated [93]. The phosphorylation of Dab1 is crucial for the activation of downstream signaling molecules, including PI3K and Crk/CrkL, which results in the modification of cytoskeleton dynamics and membrane trafficking [1, 27, 29, 57, 58, 94–97]. Thus, it would be interesting to evaluate, in the future, whether Reelin binding to LpRs triggers a signaling pathway that depends on the phosphorylation of Dab.

As previously mentioned, Reelin is not present in flies. However, a study suggests that protein domains found in vertebrate Reelin appeared in evolution with the filum Arthropoda, to which *Drosophila* belongs [98], supporting that a Reelin-like protein could exist in at least some invertebrates. We are currently analyzing the *Drosophila melanogaster* genome to unveil whether flies have a protein with a homolog function to vertebrate Reelin.

## Conclusions

We have established new physiological and developmental roles for LpR1, LpR2, and Dab in *Drosophila* MB by using heterozygous mutants and RNAi targeted expression. Overall, all these results are consistent with the idea that we have uncovered a novel signaling pathway relevant to MB formation and function. Besides, our data provide support for the existence of a Reelin-like protein in *Drosophila*.

## Methods

### Drosophila stocks

Flies strains and F1 progeny when needed were raised under 12 h:12 h light:dark cycle at 25 °C. The flies were fed with standard fly food. The flies employed were generated using flies from Bloomington Drosophila Stock Center (BDSC). Whenever needed, flies were isogenized to *w*^*1118*^ genetic background for at least five generations. Flies used were as follows: *y*^*1*^w*; Mi{PT-GFSTF.1}^*LpR2MI04745GFSTF.1 *^(RRID:BDSC_60219), LpR1-Gal4: *w*^*1118*^*; PBac{w*^+*mC*^ = *IT.GAL4}LpR1*^*0104−G4*^*/TM6B,Tb*^*1*^ (RRID:BDSC_62639), *LpR1*^*DF*^: w*;P{w^+*mC*^ = *XP-U}LpR1*^*Df*^*/TM6B,Tb*^*1*^ (balancer chromosome changed from RRID:BDSC_32653), RNAi *LpR1*: w*; P{y^+*t7.7*^* v*^+*t1.8*^ = *TRiP.JF02551}attP2 LpR1* (isogenized from RRID:BDSC_27249), *LpR2*^*DF*^: *w*^*1118*^*;rho*^*ve−1*^* PBac{w*^+*mC*^ = *RB3.WH3}LpR2*^*Df*^*/TM6B,Tb*^*1*^ (balancer chromosome changed from RRID:BDSC_44233), RNAi *LpR2*: w*; P{y^+*t7.7*^* v*^+*t1.8*^ = *TRiP.JF01627}attP2 LpR2* (isogenized from RRID:BDSC_31150), *Dab*^*1*^: *w*^*1118*^*;Dab*^*1*^*/TM6B, Tb*^*1*^ (balancer chromosome changed from RRID:BDSC_32653) and RNAi-*Dab*: w*;P{y^+*t7.7*^* v*^+*t1.8*^ = *TRiP.HMS02482}attP2 Dab* (isogenized from RRID:BDSC_42646). In addition we used the flies: *c309*-Gal4, UAS-*eGFP* (*c309,eGFP*), *Elav*-Gal4, UAS-*CD8::GFP*;;*OK107*-Gal4, *c309*-Gal4; UAS-*CD4::tdTomato.* Further, flies generated in this work include the following: the CRIMIC insertion mutant for LpR1: *yw;; LpR1 CRIMIC/TM3,Cy,* the CRIMIC insertion mutant for LpR2: *yw;; LpR2 CRIMIC/TM3, Ser*, the genomic rescue for LpR2 (*LpR2 20 Kb, BAC/SMA6a,Cy*): *yw; PBac{CH321-09A20}VK37/SM6a,Cy.*

### Measurement of mRNA levels

To demonstrate the reduction in the expression of mRNA levels in strains of interest, 50 adult male heads (0–3 days) were homogenized in TRIzol (Invitrogen). Chloroform (Merck) was added, mixed, and then centrifuged at 12,000 rpm (4 °C) for 15 min. The aqueous phase was collected and then mixed with isopropanol (Merck), followed by incubation, and posterior centrifugation at 12,000 rpm (4 °C) for 20 min. The pellet was air-dried and then resuspended in nuclease-free water at 55 °C for 15 min. RNA integrity was evaluated by running an aliquot of this preparation in an agarose gel, and RNA concentration was determined in an Epoch microplate reader (Biotek). The cDNA synthesis was carried out from 3 µg of RNA using the kit RevertAid First Strand cDNA Synthesis (Invitrogen).

The primers were directed to two exons that are present in all isoforms of the genes: LpR1: 5′–TGCACGAATGGAGCCTGCAT–3′ and 5′–GTGATGCGATCCTTGCACTGGT–3′, annealing at 60 °C; LpR2: 5′–CTATGTCGTATACCGACGCTGC–3′ and 5′–CTGCGGCGTAAATGTGTGG–3′ annealing at 57 °C; Dab: 5′–GTCTTAGCACCACGAATGGAA–3′ and 5′–GGCGTTATCCGTTCCATCT–3′ annealing at 55 °C. GAPDH was used as a reference gene: 5′–CGTTCATGCCACCACCGCTA–3′ and 5′–CCACGTCCATCACGCCACAA–3′ annealing at 60 °C. The reaction used the 5 × HOTFIREPol® EvaGreen® qPCR Mix Plus Kit (Solis BioDyne), and LightCycler® capillaries (20 μL), in the LightCycler® (Roche) apparatus. Primer efficiency was evaluated after serial dilution and was calculated as 10^−1/slope^. The relative expression was calculated as [primer efficiency for GAPDH^CT average among replica^]/ [primer efficiency for the gene of interest^CT average among replica^].

Each MB is composed of about ~ 2500 neurons [43] out of the about 100,000 found in fly brains. To test the RNAi effectiveness by qPCR, the RNAi of interest was pan-neurally driven by *Elav*-Gal4 (Additional file 1: Fig. S1c and d, Fig. S8b). This is a gal4 driver for the whole nervous system, and it is not expressed in other cell types in the fly head. Then the expression measured corresponds to an estimation of the mRNA levels.

### Aversive olfactory conditioning

Groups of 40–50 flies were collected and kept at 25 °C and 70% relative humidity under 12 h:12 h light/dark cycles in the behavioral room. Experiments were conducted using a T-maze apparatus connected to a constant airflow (3 l/min) to provide flies with either fresh air or a given odorant. The process consists of airflow passing through bubbling mineral oil or a solution of the odorant dissolved in mineral oil. The experiments were conducted under dim red light to prevent confounding visual effects on olfactory memory formation [99, 100].

On the day of the experiment, flies were transferred into a training tube lined with an electrified grid. After 90-s acclimatization to the tube, flies were exposed to an odorant (the conditioned stimulus, CS^+^), either 3-octanol (OCT) or 4-methyl cyclohexanol (MCH), paired to twelve 70 V DC electric shocks (the unconditioned stimulus, US) over 60 s (Fig. 3). This procedure was followed by a 45-s rest period with fresh air. Flies were then exposed to the reciprocal odorant (CS^−^), which is not paired to electric shock. Memory was evaluated 1 h post-training to test Mid-term memory (MTM). For this, flies are exposed to both odorants, each in one tube; the number of flies choosing each tube was quantified. A performance index (PI) was calculated as:

$$PI= \frac{({CS}^{-}-{CS}^{+})}{({CS}^{-}+{CS}^{+})}$$

The odorant used as CS^+^ was alternated between OCT and MCH in experiments carried out with different groups of flies, to account for innate bias toward one odorant. Thus, *n* = 1 corresponds to the average of the PI of two consecutive alternated groups. Sensory-motor controls were performed to assess whether avoidance of electric shocks or odorants were similar between different strains. For olfactory acuity, the odorant and air were pumped through opposite arms of the T-maze; flies were allowed to choose between the two tubes for 2 min. For electric shock avoidance, flies were allowed to decide between two training tubes, one of them connected to the stimulator providing electric shock. For olfactory and shock processing, the fly avoidance over the stimuli was quantified, and responses were reported as the percentage of flies that avoided the stimuli over the total number of flies (Additional file 1: Fig. S2).

### Locomotor and sleep analysis

Locomotor activity was measured using the Drosophila Activity Monitoring System (DAM, TriKinetics Inc, USA), using 7–9-day-old male flies. The individuals were collected, separated by phenotype, and transferred into DAM tubes containing standard fly food and placed into an incubator (25 °C and 75% humidity) connected to the monitoring system (TriKinetics, Waltham, MA, USA) under 12 h:12 h light–dark cycles for 6–7 days. After 6 days of baseline recordings, the sleep analysis was performed in R using the Rethomics framework [101]. Sleep is defined as a continuous period of inactivity lasting 5 min or more [36, 37], and activity counts to actogram profiles (Additional file 1: Fig. S2e, Fig. S3 c and f, Fig. S10d and Fig. S11c) were calculated as the number of beam crossings each 30 min.

### Fly brain immunohistochemistry

Brains from (i) *c309,eGFP*/ + ;*LpR1*^*DF*^/ + ; (ii) *c309,eGFP*/ + ;RNAi-*LpR1*/ + ; (iii) *c309,eGFP*/ + ;*LpR2*^*DF*^ / + ; and (iv) *c309,eGFP*/ + ;RNAi-*LpR2*/ + 0–3-day-old adult male flies were fixed for 20 min with 4% PFA, 4% Sucrose in PBS1X. The tissue was blocked (solution: 3% Normal Goat Serum (NGS), 0.03% Sodium Azide in Phosphate-buffered saline with 0.3% Triton-X100 (PBS-T) for 1 h, and then incubated with 1D4-FasII antibody (RRID: AB_528235) 1:20 at 4 °C, overnight (ON). After 3 h of incubation with secondary antibody Alexa Fluor 568 goat anti-mouse (RRID: AB_144696) 1:500, at room temperature (RT), brains were mounted with ProLong Gold with DAPI (Invitrogen).

### Cell culture immunocytochemistry

Primary cultured neurons were fixed with 4% paraformaldehyde (PFA) and 4% sucrose in PBS1X for 20 min. Then, cell cultures were incubated for 15 min with 0.15 M Glycine and washed three times with PBS-T. The cells were blocked (solution: 3% NGS, 0.03% Azide in PBS-T) for 1 h, followed by overnight (ON) incubation at 4° with the primary antibodies: Guinea Pig anti LpR1 (Gifted by J. Culi) 1:100 or GFP antibody (RRID: AB 94,936) 1:500. Then, the coverslips were washed three times with PBST and incubated for 2 h at RT with secondary antibodies. Secondary antibodies used were goat anti-guinea pig 648 (RRID: AB_2340476) 1:350 or Alexa Goat anti-mouse 488 (RRID: AB_2633275). Later, the cells were washed three times with PBS-T. The coverslips were mounted in Fluoromount-G with DAPI.

### Preparation of primary cultures of pupal brain neurons

As previously reported [102, 103], in brief, fly brains were dissected out of the animals at pupal stage P9, in dissecting solution (DS), consisting of 6.85 mM NaCl, 0.27 mM KCl, 0.009 mM Na2HPO4, 0.001 mM KH2PO4, 0.2772 mM HEPES, pH 7.4. After enzymatic treatment of the tissue (9.6 U/ml of papain, Worthington, LS 03,126) 30 min, at room temperature, mechanical disaggregation was carried out in culture plates containing Laminin-Concanavalin A (Sigma)-coated coverslips in the presence of DMEM/F12 culture medium (Gibco, 12,400–016) supplemented with 100 μg/ml Apo-Transferrin, 30 nM selenium, 50 μg/ml insulin, 2.1 mM 20-hydroxyecdysone, 20 ng/ml progesterone, 100 μM putrescine (all supplements from Sigma-Aldrich), and 1% antibiotic/antimycotic (Gibco 15,240,062). The following day, the cells received conditioned media obtained from astrocytes cultured in Neurobasal medium supplemented with B27 (CNBM/27).

### Reelin production

Reelin was obtained as conditioned media from a cell line (HEK293T) that stably expresses mammalian Reelin [104]. Cells were cultured in DMEM high glucose (Gibco) supplemented with fetal bovine serum (10% FBS, Biological Industries), 1% penicillin/streptomycin (Gibco), and G418 (0.5 mg/ml). In order to collect Reelin, the culture media is serum-free. The supernatant containing Reelin was collected for 5 days, and the medium was concentrated using Amicon ultra-15 100-kDa filters (Millipore). Reelin concentration in the medium was estimated semiquantitatively: BSA dilution curves and an aliquot of Reelin-containing medium were run through an SDS-PAGE. The gel was stained with Coomassie blue to visualize reelin bands. After that, the bands corresponding to BSA and Reelin were digitalized to analyze intensities in Fiji [105].

### Reelin treatments for Sholl analysis

Primary neuronal cultures were prepared on 12-mm coverslips and maintained in 24-well plates. Five days in vitro (DIV) cultures were treated with Reelin at 13 or 30 nM for 48 h. In parallel, neurons were treated with culture media collected from Hek293 cells transfected with the empty vector (Mock medium). Afterward, cultures are fixed and mounted, as mentioned above.

### Internalization assay

S2 cells were transfected with plasmids pAC5.1-LpR1J-HA, pAC5.1-LpR2E-HA, pAC5.1-LpR2F-HA, or the empty vector using the calcium phosphate method. After 48 h of expression, the cells were treated with 30 nM Reelin or the equivalent volume of Mock media for 30 min at 27 °C. Then, the cells were washed one time with Schneider medium, twice with the same medium but at pH = 3.2, and two more times with Schneider medium. The cells were fixed, and immunocytochemistry was performed as mentioned above. The primary antibodies used were HA antibody (RRID: AB_1549585) 1:500 and E4 Reelin antibody (RRID: AB_1157891) 3:17. The secondary antibodies were Alexa Fluor 488 Chicken anti Rabbit (RRID: AB_2535859) 1:500 and Alexa Fluor 568 goat anti-mouse (RRID: AB_144696) 1:500.

### Analyses

MB morphology was evaluated in both hemispheres in Fiji. It also measured the distance between the MB lobes in Fiji [105].

Statistical analyses were performed using GraphPad Prism, except for the Fisher test, which was performed using R and “fisher. test” from package “stats” version 4.0.1.

## Supplementary Information

**Additional file 1:** **Fig. S1.** Verification of reduction in LpR1 or LpR2 expression in mutant and knockdown animals. **Fig. S2.** Sensory controls in LpR1 and LpR2 mutant flies. **Fig. S3.** Knocking down LpR1 or LpR2 in MB neurons using the OK107 driver results in altered MB associated behaviors. **Fig. S4.** Reduced expression of LpR1 or LpR2 results in altered MB structure. **Fig. S5.** LpR1 expression in *w*^*1118*^ adulthood flies. **Fig. S6.** LpRs are expressed in primary culture neurons from pupal brain. **Fig. S7.** Schematic representation of LpRs isoforms studied. **Fig. S8.** Control of the Reelin internalization. **Fig. S9.** Verification of reduction in Dab expression in mutant and knockdown animals. **Fig. S10.** Sensory controls in Dab mutant flies. **Fig. S11.** Flies knockdown for Dab in the MB presents normal MB associated behaviors.

**Additional file 2:** **Table S1.** Phenotypes of flies haploinsufficient or knockout of LpR1 or LpR2 and after the re-expression of LpR2. **Table S2.** Phenotypes in insertion mutant flies for LpR1 and LpR2 and after the re-expression of LpR2. **Table S3.** Phenotypes of flies that express RNAi against LpR1 or LpR2 in MB neurons under the control of the c309-Gal4 driver. **Table S4.** Phenotypes of flies expressing RNAi against LpR1 or LpR2 in MB neurons under the control of the OK107 driver. **Table S5.** Phenotypes of mutant flies lacking a copy of Dab. **Table S6.** Phenotypes of flies that express an RNAi against Dab in MB neurons directed by c309-Gal4. **Table S7.** Phenotypes in the evaluation of genetic interaction between LpR1 and Dab.

## Data Availability

All data generated or analyzed during this study are included in this published article and its supplementary information files. The raw microscopy datasets are available from the corresponding author on reasonable request.

## Abbreviations

LpRs: Lipophorin receptors
LpR1: Lipophorin receptor 1
LpR2: Lipophorin receptor 2
MB: Mushroom bodies
LDL-R: Low-density lipoprotein receptor
ApoE: Apolipoprotein E
ApoB: Apolipoprotein B
LNv: Ventral lateral neurons
CNS: Central nervous system
Dab1: Disabled 1
CS +: Conditioned stimulus
US: Unconditioned stimulus
RAP: Receptor-associated protein
Dab: Disabled

## References

[1] Lane-Donovan C, Herz J (2017). ApoE, ApoE receptors, and the synapse in Alzheimer’s disease. Trends Endocrinol Metab.

[2] Rodenburg K, Smolenaars M, Vanhoof D, Vanderhorst D (2006). Sequence analysis of the non-recurring C-terminal domains shows that insect lipoprotein receptors constitute a distinct group of LDL receptor family members. Insect Biochem Mol Biol.

[3] Herz J, Bock HH (2002). Lipoprotein receptors in the nervous system. Annu Rev Biochem.

[4] Go G-W, Mani A (2012). Low-density Lipoprotein receptor (LDLR) Family orchestrates cholesterol Homeostasis. Yale J Biol Med.

[5] Zanoni P, Velagapudi S, Yalcinkaya M, Rohrer L, von Eckardstein A (2018). Endocytosis of lipoproteins. Atherosclerosis.

[6] Beffert U, Stolt PC, Herz J (2004). Functions of lipoprotein receptors in neurons. J Lipid Res.

[7] Dlugosz P, Nimpf J (2018). The Reelin receptors apolipoprotein E receptor 2 (ApoER2) and VLDL receptor. Int J Mol Sci.

[8] He X, Semenov M, Tamai K, Zeng X (2004). LDL receptor-related proteins 5 and 6 in Wnt/β-catenin signaling: arrows point the way. Development.

[9] Tamai K, Semenov M, Kato Y, Spokony R, Liu C, Katsuyama Y (2000). LDL-receptor-related proteins in Wnt signal transduction. Nature.

[10] Wehrli M, Dougan ST, Caldwell K, O’Keefe L, Schwartz S, Vaizel-Ohayon D (2000). arrow encodes an LDL-receptor-related protein essential for Wingless signalling. Nature.

[11] Riedel F, Vorkel D, Eaton S (2011). Megalin-dependent Yellow endocytosis restricts melanization in the Drosophila cuticle. Development.

[12] Rodenburg K, Van der Horst D (2005). Lipoprotein-mediated lipid transport in insects: Analogy to the mammalian lipid carrier system and novel concepts for the functioning of LDL receptor family members. Biochim Biophys Acta BBA - Mol Cell Biol Lipids.

[13] Parra-Peralbo E, Culi J (2011). Drosophila lipophorin receptors mediate the uptake of neutral lipids in oocytes and imaginal disc cells by an endocytosis-independent mechanism. PLoS Genet.

[14] Soukup SF, Culi J, Gubb D (2009). Uptake of the necrotic Serpin in Drosophila melanogaster via the Lipophorin Receptor-1. PLoS Genet.

[15] Huang R, Song T, Su H, Lai Z, Qin W, Tian Y (2020). High-fat diet enhances starvation-induced hyperactivity via sensitizing hunger-sensing neurons in Drosophila. eLife.

[16] Matsuo N, Nagao K, Suito T, Juni N, Kato U, Hara Y (2019). Different mechanisms for selective transport of fatty acids using a single class of lipoprotein in Drosophila. J Lipid Res.

[17] Rodríguez-Vázquez M, Vaquero D, Parra-Peralbo E, Mejía-Morales JE, Culi J. Drosophila lipophorin receptors recruit the lipoprotein LTP to the plasma membrane to mediate lipid uptake. PLOS Genet. 2015;11:e1005356. 10.1371/journal.pgen.1005356 PMC4486166 26121667

[18] Yin J, Spillman E, Cheng ES, Short J, Chen Y, Lei J, Gibbs M, Rosenthal JS, Sheng C, Chen YX, Veerasammy K (2021). Brain-specific lipoprotein receptors interact with astrocyte derived apolipoprotein and mediate neuron-glia lipid shuttling. Nat Commun.

[19] Yin J, Gibbs M, Long C, Rosenthal J, Kim HS, Kim A, Sheng C, Ding P, Javed U, Yuan Q (2018). Transcriptional regulation of lipophorin receptors supports neuronal adaptation to chronic elevations of activity. Cell Rep.

[20] Sepp KJ, Hong P, Lizarraga SB, Liu JS, Mejia LA, Walsh CA, et al. Identification of neural outgrowth genes using genome-wide RNAi. PLoS Genet. 2008;4:e1000111. 10.1371/journal.pgen.1000111 PMC2435276 18604272

[21] Andersen OM, Benhayon D, Curran T, Willnow TE (2003). Differential binding of ligands to the apolipoprotein E receptor 2. Biochemistry.

[22] Benhayon D, Magdaleno S, Curran T (2003). Binding of purified Reelin to ApoER2 and VLDLR mediates tyrosine phosphorylation of Disabled-1. Mol Brain Res.

[23] D’Arcangelo G, Homayouni R, Keshvara L, Rice DS, Sheldon M, Curran T (1999). Reelin is a ligand for lipoprotein receptors. Neuron.

[24] Yasui N, Nogi T, Takagi J (2010). Structural basis for specific recognition of Reelin by its receptors. Structure.

[25] Beffert U, Weeber EJ, Durudas A, Qiu S, Masiulis I, Sweatt JD (2005). Modulation of synaptic plasticity and memory by Reelin involves differential splicing of the lipoprotein receptor Apoer2. Neuron.

[26] D’Arcangelo GG, Miao S-C, Scares HD, Morgan JI, Curran T (1995). A protein related to extracellular matrix proteins deleted in the mouse mutant reeler. Nature.

[27] Knuesel I (2010). Reelin-mediated signaling in neuropsychiatric and neurodegenerative diseases. Prog Neurobiol.

[28] Ranaivoson FM, von Daake S, Comoletti D (2016). Structural insights into Reelin function: present and future. Front Cell Neurosci.

[29] Santana J, Marzolo M-P (2017). The functions of Reelin in membrane trafficking and cytoskeletal dynamics: implications for neuronal migration, polarization and differentiation. Biochem J.

[30] Trommsdorff M, Gotthardt M, Hiesberger T, Shelton J, Stockinger W, Nimpf J (1999). Reeler/disabled-like disruption of neuronal migration in knockout mice lacking the VLDL receptor and ApoE receptor 2. Cell.

[31] Weeber EJ, Beffert U, Jones C, Christian JM, Förster E, Sweatt JD (2002). Reelin and ApoE receptors cooperate to enhance hippocampal synaptic plasticity and learning. J Biol Chem.

[32] Howell BW (1997). Mouse disabled (mDab1): a Src binding protein implicated in neuronal development. EMBO J.

[33] Kawasaki F, Iyer J, Posey LL, Sun CE, Mammen SE, Yan H (2011). The DISABLED protein functions in CLATHRIN-mediated synaptic vesicle endocytosis and exoendocytic coupling at the active zone. Proc Natl Acad Sci.

[34] Yun M, Keshvara L, Park C-G, Dickerson JB, Zheng J, Rock CO (2003). Crystal structures of the Dab homology domains of mouse disabled 1 and 2. J Biol Chem.

[35] Hiesberger T, Trommsdorff M, Howell BW, Goffinet A, Mumby MC, Cooper JA (1999). Direct binding of Reelin to VLDL receptor and ApoE receptor 2 induces tyrosine phosphorylation of disabled-1 and modulates tau phosphorylation. Neuron.

[36] Hendricks JC, Finn SM, Panckeri KA, Chavkin J, Williams JA, Sehgal A (2000). Rest in Drosophila is a sleep-like state. Neuron.

[37] Shaw PJ (2000). Correlates of sleep and waking in Drosophila melanogaster. Science.

[38] Haynes PR, Christmann BL, Griffith LC (2015). A single pair of neurons links sleep to memory consolidation in Drosophila melanogaster. ELife.

[39] Martin J-R, Ernst R, Heisenberg M (1998). Mushroom bodies suppress locomotor activity in Drosophila melanogaster. Learn Mem.

[40] Pitman JL, McGill JJ, Keegan KP, Allada R (2006). A dynamic role for the mushroom bodies in promoting sleep in Drosophila. Nature.

[41] Sitaraman D, Aso Y, Jin X, Chen N, Felix M, Rubin GM (2015). Propagation of homeostatic sleep signals by segregated synaptic microcircuits of the Drosophila Mushroom Body. Curr Biol.

[42] Vogt K, Schnaitmann C, Dylla KV, Knapek S, Aso Y, Rubin GM (2014). Shared mushroom body circuits underlie visual and olfactory memories in Drosophila. eLife.

[43] Aso Y, Grübel K, Busch S, Friedrich AB, Siwanowicz I, Tanimoto H (2009). The Mushroom body of adult Drosophila characterized by GAL4 drivers. J Neurogenet.

[44] Heisenberg M (2003). Mushroom body memoir: from maps to models. Nat Rev Neurosci.

[45] Crittenden JR, Skoulakis EMC, Han K-A, Kalderon D, Davis RL (1998). Tripartite mushroom body architecture revealed by antigenic markers. Learn Mem.

[46] Huang C, Zheng X, Zhao H, Li M, Wang P, Xie Z (2012). A permissive role of mushroom body α/β core neurons in long-term memory consolidation in Drosophila. Curr Biol.

[47] Pascual A (2001). Localization of long-term memory within the Drosophila mushroom body. Science.

[48] Zwarts L, Vanden Broeck L, Cappuyns E, Ayroles JF, Magwire MM, Vulsteke V (2015). The genetic basis of natural variation in mushroom body size in Drosophila melanogaster. Nat Commun.

[49] Connolly JB, Roberts IJH, Armstrong JD, Kaiser K, Forte M, Tully T (1996). Associative learning disrupted by impaired Gs signaling in Drosophila Mushroom Bodies. Science.

[50] Martín-Peña A, Acebes A, Rodríguez J-R, Chevalier V, Casas-Tinto S, Triphan T (2014). Cell types and coincident synapses in the ellipsoid body of *Drosophila*. Eur J Neurosci.

[51] Omoto JJ, Nguyen B-CM, Kandimalla P, Lovick JK, Donlea JM, Hartenstein V (2018). Neuronal constituents and putative interactions within the Drosophila ellipsoid body neuropil. Front Neural Circuits.

[52] Zhang Z, Li X, Guo J, Li Y, Guo A (2013). Two clusters of GABAergic ellipsoid body neurons modulate olfactory labile memory in Drosophila. J Neurosci.

[53] Nagarkar-Jaiswal S, Lee P-T, Campbell ME, Chen K, Anguiano-Zarate S, Cantu Gutierrez M (2015). A library of MiMICs allows tagging of genes and reversible, spatial and temporal knockdown of proteins in Drosophila. eLife.

[54] Kunz T, Kraft KF, Technau GM, Urbach R (2012). Origin of Drosophila mushroom body neuroblasts and generation of divergent embryonic lineages. Development.

[55] Lee T, Lee A, Luo L (1999). Clonal analysis of the mushroom bodies. Development.

[56] D’Arcangelo G (2014). Reelin in the years: controlling neuronal migration and maturation in the mammalian brain. Adv Neurosci.

[57] Jossin Y, Goffinet AM (2007). Reelin Signals through phosphatidylinositol 3-kinase and Akt to control cortical development and through mTor to regulate dendritic growth. Mol Cell Biol.

[58] Matsuki T, Pramatarova A, Howell BW (2008). Reduction of Crk and CrkL expression blocks reelin-induced dendritogenesis. J Cell Sci.

[59] Niu S, Renfro A, Quattrocchi CC, Sheldon M, D’Arcangelo G (2004). Reelin promotes hippocampal dendrite development through the VLDLR/ApoER2-Dab1 pathway. Neuron.

[60] Sotelo P, Farfán P, Benitez ML, Bu G, Marzolo M-P (2014). Sorting Nexin 17 regulates ApoER2 recycling and Reelin signaling. Wanjin H, editor. PLoS One.

[61] Ampuero E, Jury N, Härtel S, Marzolo M-P, van Zundert B (2017). Interfering of the Reelin/ApoER2/PSD95 signaling axis reactivates dendritogenesis of mature hippocampal neurons: Reelin signaling maintains adult network stability. J Cell Physiol.

[62] Bu G, Marzolo MP (2000). Role of RAP in the biogenesis of lipoprotein receptors. Trends Cardiovasc Med.

[63] Battey FD, Gåfvels ME, FitzGerald DJ, Argraves WS, Chappell DA, Strauss JF (1994). The 39-kDa receptor-associated protein regulates ligand binding by the very low density lipoprotein receptor. J Biol Chem.

[64] Stockinger W, Hengstschläger-Ottnad E, Novak S, Matus A, Hüttinger M, Bauer J (1998). The low density lipoprotein receptor gene family. J Biol Chem.

[65] Van Hoof D (2002). Insect lipoprotein follows a transferrin-like recycling pathway that is mediated by the insect LDL receptor homologue. J Cell Sci.

[66] Keshvara L, Benhayon D, Magdaleno S, Curran T (2001). Identification of Reelin-induced sites of tyrosyl phosphorylation on disabled 1. J Biol Chem.

[67] Howell BW, Hawkes R, Soriano P, Cooper JA (1997). Neuronal position in the developing brain is regulated by mouse disabled-1. Nature.

[68] Song JK, Kannan R, Merdes G, Singh J, Mlodzik M, Giniger E (2010). Disabled is a bona fide component of the Abl signaling network. Development.

[69] McGuire SE (2001). The role of Drosophila mushroom body signaling in olfactory memory. Science.

[70] Hourcade B, Muenz TS, Sandoz JC, Rossler W, Devaud JM (2010). Long-term memory leads to synaptic reorganization in the mushroom bodies: a memory trace in the insect brain?. J Neurosci.

[71] Maza FJ, Sztarker J, Shkedy A, Peszano VN, Locatelli FF, Delorenzi A (2016). Context-dependent memory traces in the crab’s mushroom bodies: functional support for a common origin of high-order memory centers. Proc Natl Acad Sci.

[72] Owald D, Waddell S (2015). Olfactory learning skews mushroom body output pathways to steer behavioral choice in Drosophila. Curr Opin Neurobiol.

[73] Wolff GH, Strausfeld NJ (2016). Genealogical correspondence of a forebrain centre implies an executive brain in the protostome–deuterostome bilaterian ancestor. Philos Trans R Soc B Biol Sci.

[74] Whited JL, Robichaux MB, Yang JC, Garrity PA (2007). Ptpmeg is required for the proper establishment and maintenance of axon projections in the central brain of Drosophila. Development.

[75] Jia M, Meng D, Chen M, Li T, Zhang YQ, Yao A (2019). Drosophila homolog of the intellectual disability-related long-chain acyl-CoA synthetase 4 is required for neuroblast proliferation. J Genet Genomics.

[76] Stoeckli ET (2018). Understanding axon guidance: are we nearly there yet?. Development.

[77] Faini G, Del Bene F, Albadri S (2021). Reelin functions beyond neuronal migration: from synaptogenesis to network activity modulation. Curr Opin Neurobiol.

[78] Del Río JA, Heimrich B, Borrell V, Förster E, Drakew A, Alcántara S (1997). A role for Cajal-Retzius cells and reelin in the development of hippocampal connections. Nature.

[79] Di Donato V, De Santis F, Albadri S, Auer TO, Duroure K, Charpentier M (2018). An attractive reelin gradient establishes synaptic lamination in the vertebrate visual system. Neuron.

[80] Rice DS, Nusinowitz S, Azimi AM, Martínez A, Soriano E, Curran T (2001). The Reelin pathway modulates the structure and function of retinal synaptic circuitry. Neuron.

[81] Borrell V, Del Río JA, Alcántara S, Derer M, Martínez A, D’Arcangelo G (1999). Reelin regulates the development and synaptogenesis of the layer-specific entorhino-hippocampal connections. J Neurosci.

[82] Borrell V, Pujadas L, Simó S, Durà D, Solé M, Cooper JA (2007). Reelin and mDab1 regulate the development of hippocampal connections. Mol Cell Neurosci.

[83] Chai X, Förster E, Zhao S, Bock HH, Frotscher M (2009). Reelin acts as a stop signal for radially migrating neurons by inducing phosphorylation of n-cofilin at the leading edge. Commun Integr Biol.

[84] Ng J (2008). TGFβ signals regulate axonal development through distinct Smad-independent mechanisms. Development.

[85] Ng J, Luo L (2004). Rho GTPases regulate axon growth through convergent and divergent signaling pathways. Neuron.

[86] Ng J, Nardine T, Harms M, Tzu J, Goldstein A, Sun Y (2002). Rac GTPases control axon growth, guidance and branching. Nature.

[87] Boyle M, Nighorn A, Thomas JB (2006). Drosophila Eph receptor guides specific axon branches of mushroom body neurons. Development.

[88] Reynaud E, Lahaye LL, Boulanger A, Petrova IM, Marquilly C, Flandre A (2015). Guidance of Drosophila mushroom body axons depends upon DRL-Wnt receptor cleavage in the brain dorsomedial lineage precursors. Cell Rep.

[89] Heisenberg M, Borst A, Wagner S, Byers D (1985). The mushroom body mutants are deficient in olfactory learning. J Neurogenet.

[90] Kosacka J, Gericke M, Nowicki M, Kacza J, Borlak J, Spanel-Borowski K (2009). Apolipoproteins D and E3 exert neurotrophic and synaptogenic effects in dorsal root ganglion cell cultures. Neuroscience.

[91] Liu L, MacKenzie KR, Putluri N, Maletić-Savatić M, Bellen HJ (2017). The glia-neuron lactate shuttle and elevated ROS promote lipid synthesis in neurons and lipid droplet accumulation in glia via APOE/D. Cell Metab.

[92] Howell BW, Lanier LM, Frank R, Gertler FB, Cooper JA (1999). The disabled 1 phosphotyrosine-binding domain binds to the internalization signals of transmembrane glycoproteins and to phospholipids. Mol Cell Biol.

[93] Gertler FB, Hill KK, Clark MJ, Hoffmann FM (1993). Dosage-sensitive modifiers of Drosophila abl tyrosine kinase function: prospero, a regulator of axonal outgrowth, and disabled, a novel tyrosine kinase substrate. Genes Dev.

[94] Beffert U, Morfini G, Bock HH, Reyna H, Brady ST, Herz J (2002). Reelin-mediated signaling locally regulates protein kinase B/Akt and glycogen synthase kinase 3β. J Biol Chem.

[95] Chen K (2004). Interaction between Dab1 and CrkII is promoted by Reelin signaling. J Cell Sci.

[96] Howell BW, Herrick TM, Hildebrand JD, Zhang Y, Cooper JA (2000). Dab1 tyrosine phosphorylation sites relay positional signals during mouse brain development. Curr Biol.

[97] Leemhuis J, Bouche E, Frotscher M, Henle F, Hein L, Herz J (2010). Reelin signals through apolipoprotein E receptor 2 and Cdc42 to increase growth cone motility and filopodia formation. J Neurosci.

[98] Manoharan M, Muhammad SA, Sowdhamini R (2015). Sequence analysis and evolutionary studies of Reelin proteins. Bioinforma Biol Insights.

[99] Tully T, Quinn WG (1985). Classical conditioning and retention in normal and mutant Drosophila melanogaster. J Comp Physiol [A].

[100] Hidalgo S, Campusano JM, Hodge JJL (2021). Assessing olfactory, memory, social and circadian phenotypes associated with schizophrenia in a genetic model based on Rim. Transl Psychiatry.

[101] Geissmann Q, Garcia Rodriguez L, Beckwith EJ, Gilestro GF (2019). Rethomics: An R framework to analyse high-throughput behavioural data. Flack JC, editor. PLOS ONE..

[102] Leyton V, Goles NI, Fuenzalida-Uribe N, Campusano JMV (2014). Octopamine and Dopamine differentially modulate the nicotine-induced calcium response in Drosophila mushroom body kenyon cells. Neurosci Lett.

[103] Sicaeros B, Campusano JM, O’Dowd DK. Primary neuronal cultures from the brains of late stage Drosophila pupae. J Vis Exp. 2007;4:e200. 10.3791/200 PMC2556163 18979004

[104] D’Arcangelo G, Nakajima K, Miyata T, Ogawa M, Mikoshiba K, Curran T (1997). Reelin is a secreted glycoprotein recognized by the CR-50 monoclonal antibody. J Neurosci.

[105] Schindelin J, Arganda-Carreras I, Frise E, Kaynig V, Longair M, Pietzsch T (2012). Fiji: an open-source platform for biological-image analysis. Nat Methods.

