# Supplementary material for: Lipophorin receptors regulate mushroom body development and complex behaviors in Drosophila
Source: BMC Biol. 2022 Sep 7;20:198. doi: 10.1186/s12915-022-01393-1 (PMC9454125; doi:10.1186/s12915-022-01393-1)
Supplement: Supplementary file 1 — Additional file 1: Fig. S1. Verification of reduction in LpR1 or LpR2 expression in mutant and knockdown animals. Fig. S2. Sensory controls in LpR1 and LpR2 mutant flies. Fig. S3. Knocking down LpR1 or LpR2 in MB neurons using the OK107 driver results in altered MB associated behaviors. Fig. S4. Reduced expression of LpR1 or LpR2 results in altered MB structure. Fig. S5. LpR1 expression in w1118 adulthood flies. Fig. S6. LpRs are expressed in primary culture neurons from pupal brain. Fig. S7. Schematic representation of LpRs isoforms studied. Fig. S8. Control of the Reelin internalization. Fig. S9. Verification of reduction in Dab expression in mutant and knockdown animals. Fig. S10. Sensory controls in Dab mutant flies. Fig. S11. Flies knockdown for Dab in the MB presents normal MB associated behaviors. [file 12915_2022_1393_MOESM1_ESM.pdf]

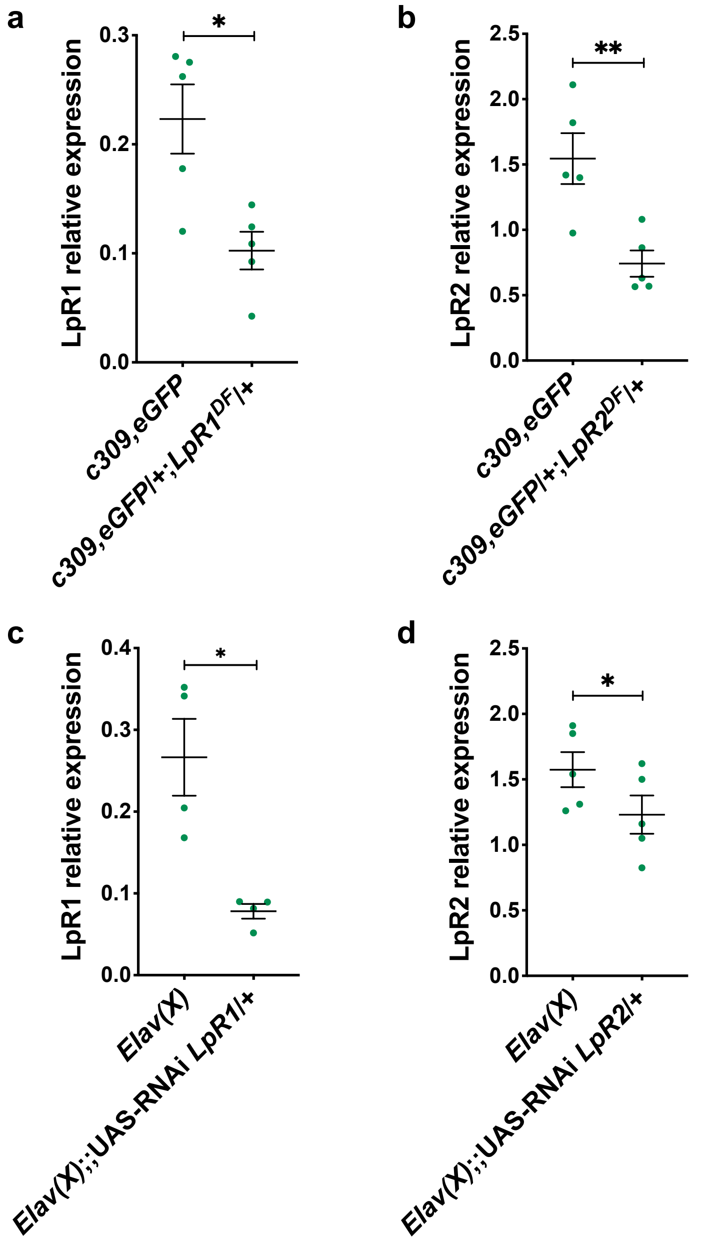


**Fig. S1 Verification of reduction in LpR1 or LpR2 expression in mutant and knockdown animals**. **a-b** Reduced expression of LpR1 or LpR2 in flies heterozygous for deletion in *LpR1* or *LpR2* genes (*c309,eGFP*/+; *LpR1*$^{DF}$/+ and *c309,eGFP*/+;*LpR2*$^{DF}$/+, respectively) evaluated through qPCR. Data from n=5 independent experiments for each genotype; * and ** means significant differences after paired t-test, p=0.0128 and p=0.0018, respectively. **c-d** Reduced LpR1 and LpR2 expression in flies expressing RNAi for each transcript (*Elav(x);;*UAS-RNAi *LpR1* and *Elav(x)*;;UAS-RNAi *LpR2*) in the whole nervous system by using the Elav driver. Data from n=4 and 5 independent experiments, respectively; * significant differences after paired t-test (p=0.0391 and p=0.0439).

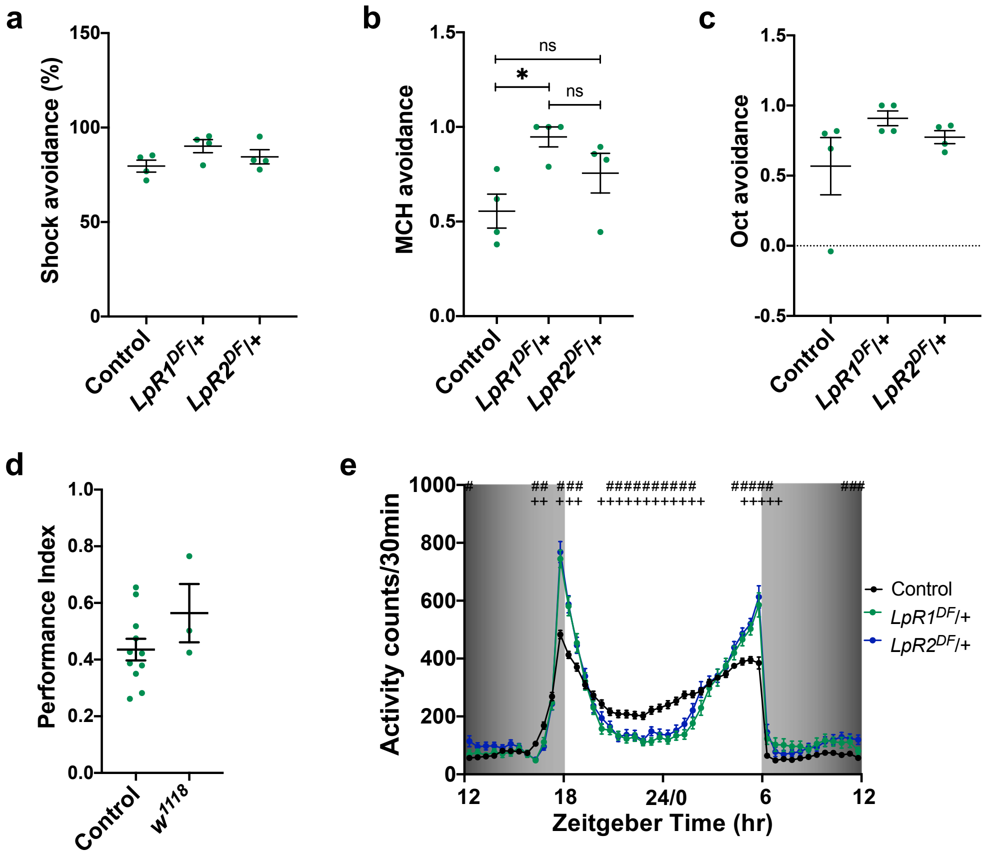


**Fig. S2 Sensory controls in LpR1 and LpR2 mutant flies. a** Response to electric shocks in flies that lack a copy of LpR1 or LpR2 compared to control. Data expressed as mean ± SEM; n=4 independent experiments, one-way ANOVA P=0.9687, no differences between groups. **b** Innate response to MCH. Data expressed as mean ± SEM of n=4 independent experiments, one-way ANOVA (P=0.0201) followed by Kruskal Wallis test. Flies mutant for LpR1 exhibit a higher sensitivity to MCH than controls; * P<0.05, "ns" no significant differences. **c** Innate response to Octanol (Oct). Data expressed as mean ± SEM from n=4 independent experiments. One-way ANOVA shows no differences between groups (p=0.1082). **d** Comparison in performance index in Cs$^{w-}$ and $w^{1118}$ flies; t-test p=0.1743, no differences between genotypes. Data expressed as mean ± SEM from n= 11 or 3 independent experiments, respectively. **e** Locomotor activity profile throughout the day in flies mutant for LpR1 or LpR2. Data from n=2 independent experiments, 15-25 flies studied per genotype in each n. Data expressed as mean ± SEM. Two-way ANOVA, Tukey post-test, shows that the hour of the day, genotype factors, and the interaction between factors contribute to results (p<0.0001 for each analysis). "+" indicates a significant difference (p<0.05) between Control fly and mutant for LpR1, at a given time of the day; "#", a significant difference (p<0.05) between Control and LpR2 mutant flies at the same hour of the day.

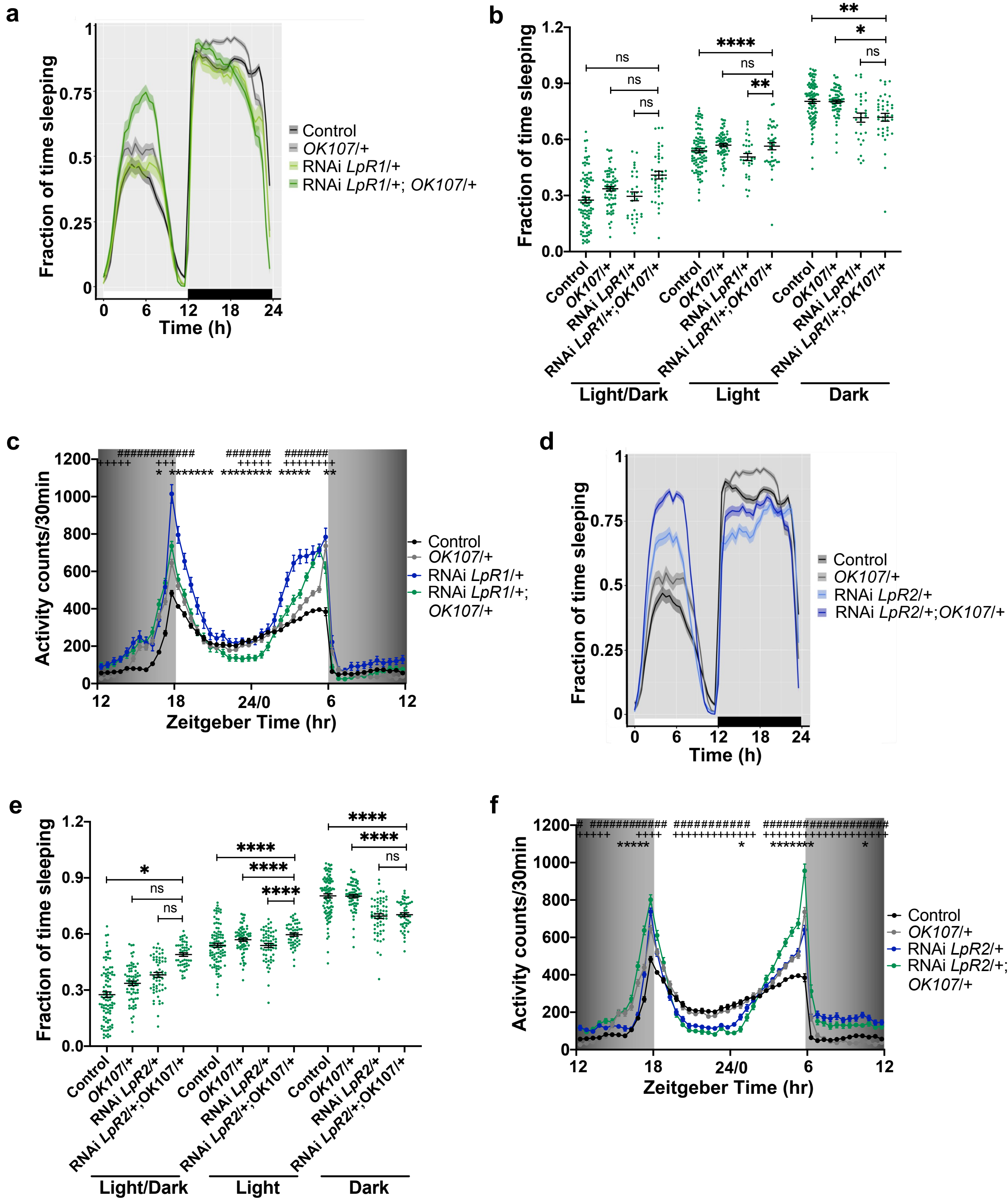
a
Fraction of time sleeping
1
0.75
0.5
0.25
0
0
6
12
18
24
Time (h)
Control
OK107/+
RNAi LpR1/+
RNAi LpR1/+; OK107/+
b
Fraction of time sleeping
1.2
0.9
0.6
0.3
0.0
ns
ns
ns
****
ns
**
**
*
ns
Control
OK107/+
RNAi LpR1/+
RNAi LpR1/+;OK107/+
Light/Dark
Light
Dark
c
Activity counts/30min
1200
1000
800
600
400
200
0
12
18
24/0
6
12
Zeitgeber Time (hr)
Control
OK107/+
RNAi LpR1/+
RNAi LpR1/+; OK107/+
d
Fraction of time sleeping
1
0.75
0.5
0.25
0
0
6
12
18
24
Time (h)
Control
OK107/+
RNAi LpR2/+
RNAi LpR2/+;OK107/+
e
Fraction of time sleeping
1.2
0.9
0.6
0.3
0.0
*
ns
ns
****
****
****
****
****
ns
Control
OK107/+
RNAi LpR2/+
RNAi LpR2/+;OK107/+
Light/Dark
Light
Dark
f
Activity counts/30min
1200
1000
800
600
400
200
0
12
18
24/0
6
12
Zeitgeber Time (hr)
Control
OK107/+
RNAi LpR2/+
RNAi LpR2/+; OK107/+

**Fig. S3 Knocking down LpR1 or LpR2 in MB neurons using the OK107 driver results in altered MB associated behaviors. a** Sleep profile in flies that express a RNAi against LpR1 in the MB. **b** Fraction of time sleeping in flies knockdown for LpR1 in the MB. Data in **a**-**c**, from two independent experiments, n=35-95 flies studied per genotype. **c** Locomotor activity profile throughout the day in flies expressing an RNAi against LpR1. Two-way ANOVA, Tukey post test, show that the hour of the day, and genotype factors, and also interaction between factors, play a role in results ($p<0.0001$). "+" indicates significant difference ($p<0.05$) between *OK107*/+ control fly and flies expressing a RNAi against LpR1 in the MB (RNAi *LpR1*/+;*OK107*/+ ), at a given time of the day; "#", significant difference ($p<0.05$) between CS control flies and the knockdown for LpR1 in the MB at the same hour of the day. "*" indicates significant difference ($p<0.05$) between RNAi *LpR1*/+ control fly and flies expressing a RNAi against LpR1 in the MB at a given time of the day. **d** Sleep profile in flies expressing an RNAi against LpR2 in the MB. **e** Fraction of time sleeping in flies knockdown for LpR2 in the MB. **f** Locomotor activity profile throughout the day in flies expressing an RNAi against LpR2. Two-way ANOVA, Tukey post test, show that the hour of the day, and genotype factors, and also interaction between factors, play a role in results ($p<0.0001$). "+" indicates significant difference ($p<0.05$) between *OK107*/+ control fly and flies expressing a RNAi against LpR2 in the MB (RNAi *LpR2*/+;*OK107*/+), at a given time of the day; "#", significant difference ($p<0.05$) between CS control flies and the knockdown for LpR2 in the MB at the same hour of the day. "*" indicates significant difference ($p<0.05$) between RNAi *LpR2*/+ control fly and flies knockdown for LpR2 in the MB at a given time of the day. Data in **d**-**f**, from two independent experiments, n=50-95 flies studied per genotype. In **a** and **d** lower white left bar represents the hours of the day where flies were exposed to light. Lower black right bar represents the hours of the day were fly were exposed to darkness. **a**-**f** Data expressed as mean ± SEM. In **b** and **e** two-way ANOVA show that light and genotype factors, and also interaction between factors, play a role in results ($p<0.0001$ for each analysis), Tukey post test; *, ** and ****, indicates $p<0.05$, $p<0.01$ and $p<0.0001$ between conditions. ns, not significant. Control strain in these experiments is Canton-S.

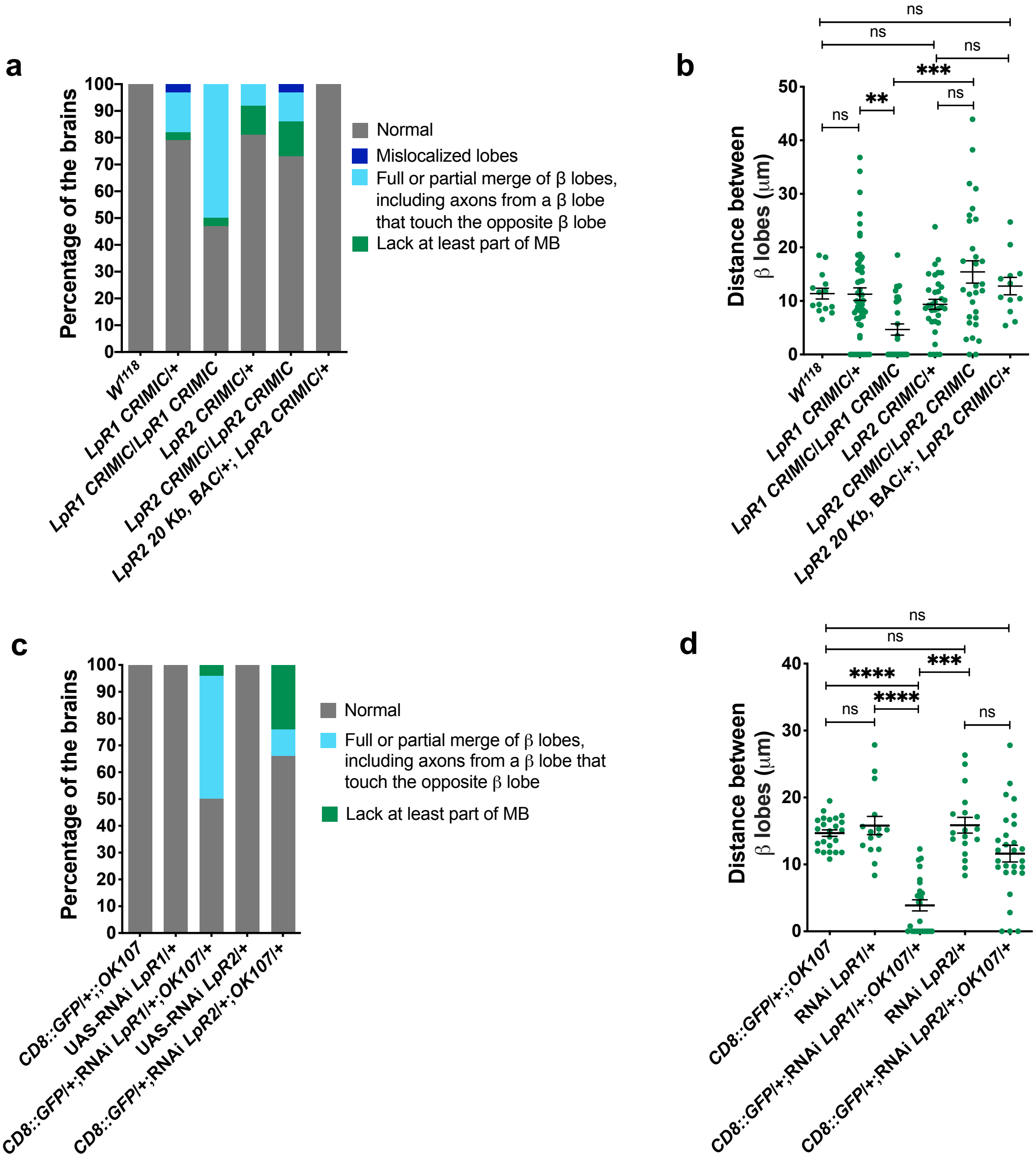
a
Percentage of the brains
Normal
Mislocalized lobes
Full or partial merge of β lobes, including axons from a β lobe that touch the opposite β lobe
Lack at least part of MB
W1118
LpR1 CRIMIC/+
LpR1 CRIMIC/LpR1 CRIMIC
LpR2 CRIMIC/+
LpR2 CRIMIC/LpR2 CRIMIC
LpR2 20 Kb, BAC/+; LpR2 CRIMIC/+
b
Distance between β lobes (μm)
ns
**
***
c
Normal
Full or partial merge of β lobes, including axons from a β lobe that touch the opposite β lobe
Lack at least part of MB
CD8::GFP/+;;OK107
UAS-RNAi LpR1/+
CD8::GFP/+;RNAi LpR1/+;OK107/+
UAS-RNAi LpR2/+
CD8::GFP/+;RNAi LpR2/+;OK107/+
d
****
RNAi LpR1/+
RNAi LpR2/+

**Fig. S4 Reduced expression of LpR1 or LpR2 results in altered MB structure. a** Percentage of adulthood brains that exhibit different phenotypes in CRIMIC mutants and deletion mutant for LpR2. Data from $w^{1118}$ (n=2 experiments, 14 brains altogether); *LpR1 CRIMIC*/+ (n=4 experiments, 57 brains); *LpR1 CRMIC*/*LpR1 CRIMIC* (n=2 experiments, 30 brains); *LpR2 CRIMIC*/+ (n=2 experiments, 37 brains); *LpR2 CRIMIC*/*LpR2 CRIMIC.* (n=2 experiments, 30 brains); *LpR2 20Kb,* BAC/+; *LpR2 CRIMIC*/+ (n=1 experiments, 12 brains); *c309,eGFP* control animals (n=4 experiments, 40 brains altogether); *c309,eGFP*/+;$LpR2^{DF}$/+ (n=4 experiments, 46 brains altogether); *c309,eGFP*/*LpR2 20Kb,* BAC;$LpR2^{DF}$/+ (n=1 experiments, 23 brains altogether). Fisher test for $w^{1118}$*, LpR1 CRIMIC/+, LpR1 CRIMIC/LpR1 CRIMIC* p=2.2*10-16. Fisher test for $w^{1118}$*, LpR2 CRIMIC/+, LpR2 CRIMIC/LpR2 CRIMIC, LpR2 20Kb,* BAC/+; *LpR2 CRIMIC*/+ p=1.2*$10^{-12}$. See also Table S2. **b** Distance between β lobes in CRIMIC mutants for LpRs and the genomic rescue for LpR2. Data from $W^{1118}$ (n=2 experiments, 14 brains); *LpR1 CRIMIC*/+ (n=4 experiments, 57 brains); *LpR1 CRMIC*/*LpR1 CRIMIC* (n=2 experiments, 30 brains); *LpR2 CRIMIC*/+ (n=2 experiments, 35 brains); *LpR2 CRIMIC*/*LpR2 CRIMIC.* (n=2 experiments, 30 brains); *LpR2 20Kb,* BAC/+; *LpR2 CRIMIC*/+ (n=1 experiments, 12 brains). One-way ANOVA (p=0.0002) followed by Kruskal Wallis.** and **** means p=0.0073 and p=0.0002 respectively. “ns” means not statically significant. **c** Percentage of brains that exhibit different phenotypes after expression of RNAi for LpR1 or LpR2 in MB, driven by OK107-Gal4. Data from *CD8::GFP*/+;;*OK107* flies (n=3 experiments, 25 brains); *RNAi LpR1*/+ flies (n=1 experiments, 15 brains); *CD8::GFP*/+;*RNAi LpR1*/+;*OK107*/+ animals (n=3 experiments, 24 brains); *RNAi LpR2*/+ flies (n=1 experiments, 18 brains); CD8::GFP/+;*RNAi LpR2*/+;*OK107*/+ flies (n=3 experiments, 29 brains). Fisher test for *OK107,* RNAi *LpR1/+,* RNAi *LpR1/+;;OK107/+* P=2.2*$10^{-16}$. Fisher test for *OK107,* RNAi *LpR2/+,* RNAi *LpR2/+;;OK107/+* P=2.2*$10^{-16}$. See also Table S4. **d** Distance between β lobes after knocking down LpR1 or LpR2 in the MB by using the OK107 driver. Data expressed as mean ± SEM from *CD8::GFP*/+;;*OK107* (n=3 experiments, 26 brains); *RNAi LpR1*/+ flies (n=1 experiments, 15 brains); *CD8::GFP*/+;*RNAi LpR1*/+;*OK107*/+ (n=3 experiments, 26 brains); *RNAi LpR2*/+ flies (n=1 experiments, 18 brains); *CD8::GFP*/+;*RNAi LpR2*/+;*OK107*/+ (n=3 experiments, 29 brains) flies. One-way ANOVA (p<0.0001) followed by Kruskal Wallis. **, **** means p<0.0014 and p<0.0001 respectively. “ns” means not statically significant.

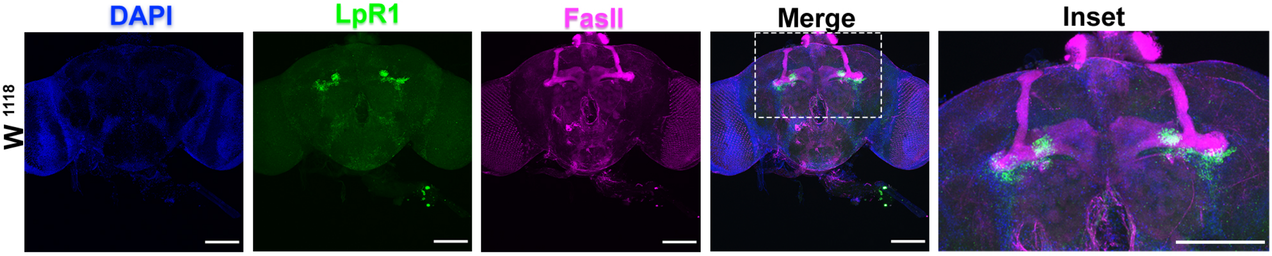


**Fig. S5 LpR1 expression in w1118 adulthood flies.** Representative image of immunofluorescence against LpR1 and FasII in w1118 flies with DAPI staining. The discontinuous line in the merge indicates the magnified region in the inset. Scale white bars represent 50 µm.

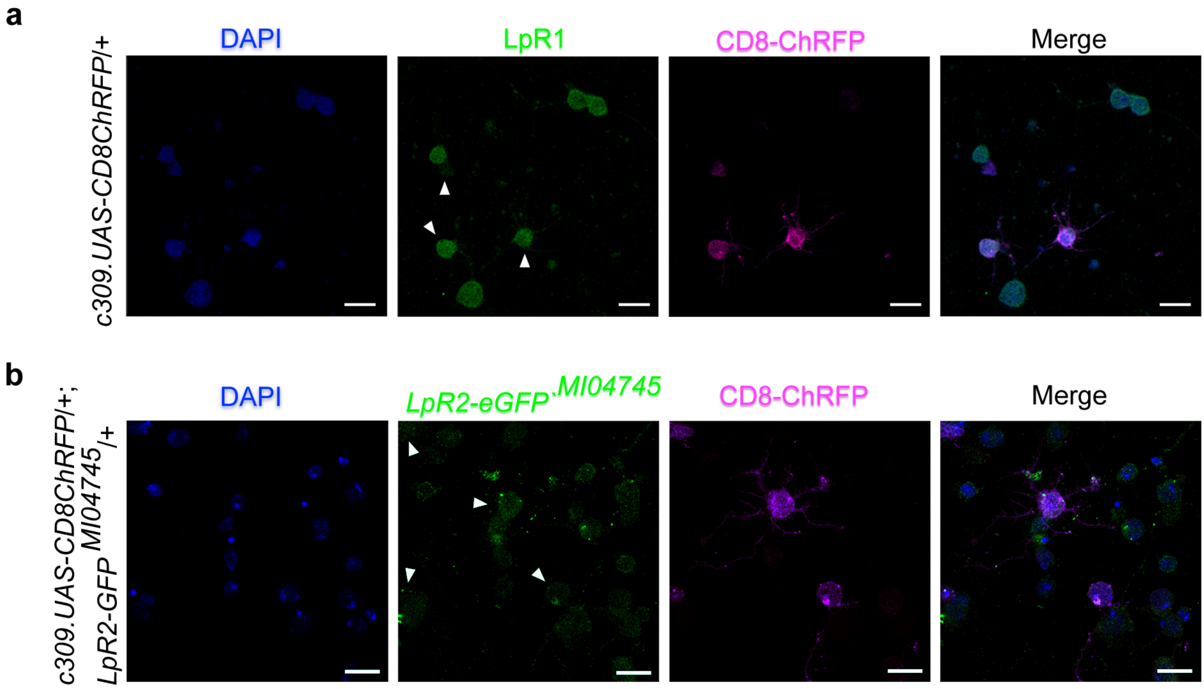


**Fig. S6 LpRs are expressed in primary culture neurons from pupal brain. a** Representative results of immunofluorescence experiment to detect LpR1 in primary cultures of a Drosophila brain where MB neurons express the membrane protein CD8-ChRFP under the control of the c309-Gal4 driver (c309-Gal4>UAS-CD8.ChRFP). Arrowheads show MB-positive cells identified by CD8.ChRFP expression. **b** Representative images of immunofluorescence staining for GFP in primary cultures of a Drosophila brain expressing LpR2-GFPMI04745 where MB neurons are identified by CD8.ChRFP expression. Arrowheads indicate MB-positive cells. The white bar represents 5μm.

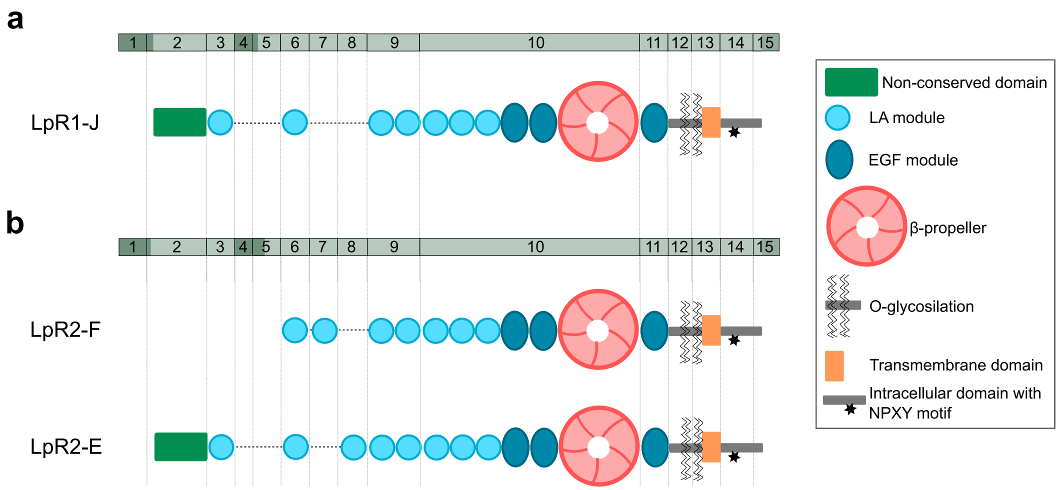


**Fig. S7 Schematic representation of LpRs isoforms studied.** Organization of the domains in the LpRs isoforms studied with its correspondence exons for LpR1 (**a**) and LpR2 (**b**). At the top of each panel, the green box represents the exons labels 1-15. The dark green mark indicates the UTR regions for each cDNA.

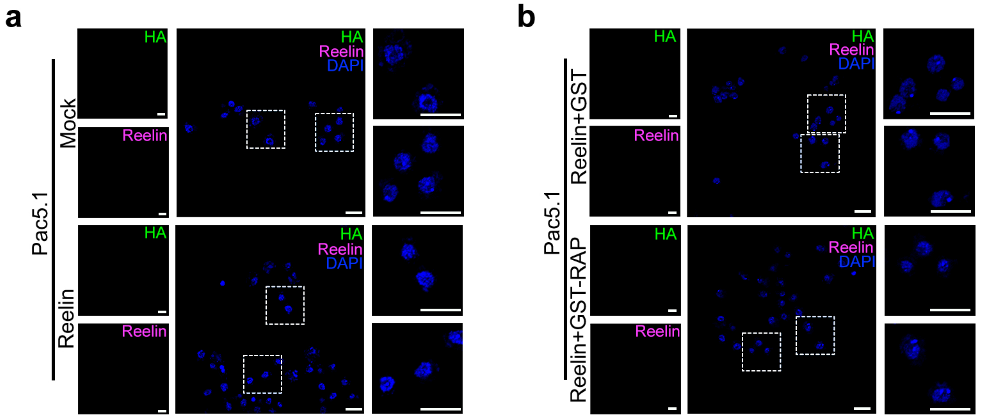


**Fig. S8 Control of the Reelin internalization. a** Representative images of immunocytochemistry of anti-HA and Reelin in S2 cells transiently transfected with the empty vector pAC5.1 which were treated with 30nM Reelin or the equivalent volume of Mock media under the same internalization protocol as in Fig 6A. **b** Representative images of immunocytochemistry of anti-HA and Reelin in S2 cells transiently transfected with the empty vector pAC5.1 which were treated with 30nM Reelin plus 500nM of GST-RAP or 500nM GST under the same internalization protocol used in Fig 6b**.**

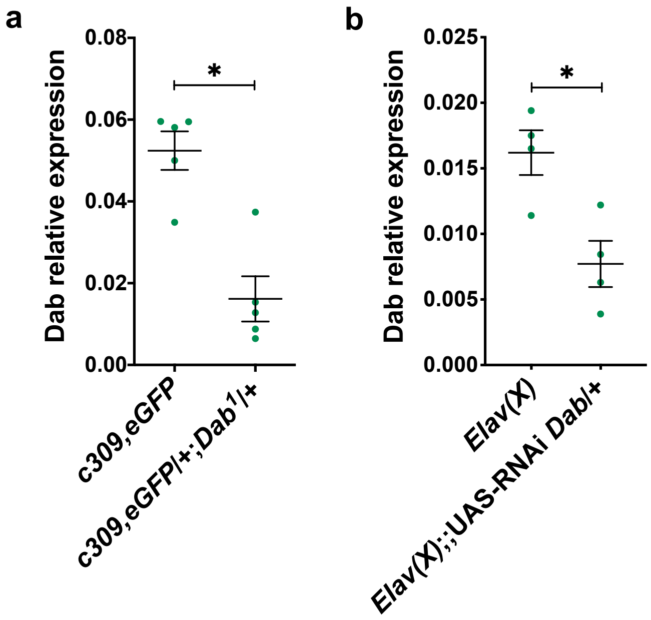


**Fig. S9 Verification of reduction in Dab expression in mutant and knockdown animals. a** Reduced expression of Dab in mutant *c309,eGFP*/+;*Dab*$^{1}$/+ flies lacking one copy of the gene evaluated through qPCR. Data expressed as mean ± SEM; paired t-test, * p=0.0105. Data from n=5 independent experiments, 50 fly heads each "n". **b** Expressing RNAi for Dab pan-neurally (*Elav(x)*/+;;UASRNAi *Dab*/+) results in decreased expression of transcript for the protein. Data from n=4 independent experiments for each genotype, 50 fly heads each "n". * means statistical differences after paired t-test, p=0.0141.

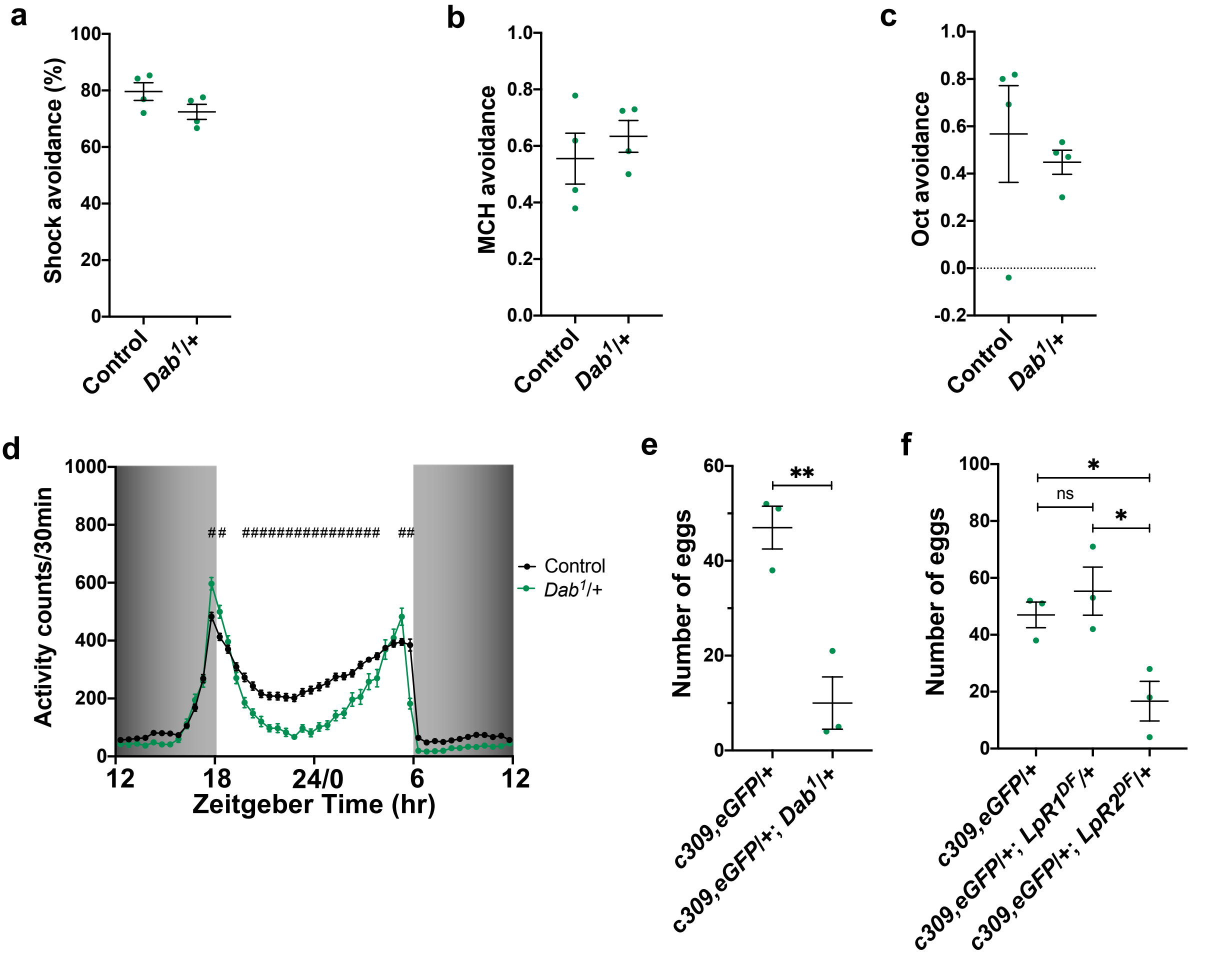


**Fig. S10 Sensory controls in Dab mutant flies. a** Shock avoidance in Dab mutants (*$Dab^1$*/+). Data expressed as mean ± SEM of n=4 independent experiments; t-test p=0.1331, no significant differences. **b**-**c** Innate response to MCH and Oct, respectively, in mutant *$Dab^1$*/+ flies compared to controls. Data expressed as mean ± SEM of n=4 independent experiments; t-test p=0.4860 and p=0.3429, no differences between groups. **d** Locomotor activity profile throughout the day in flies mutant for *Dab*. Data from n=2 independent experiments, 15-25 flies studied per genotype in each n. Data expressed as mean ± SEM. Two-way ANOVA, Tukey post-test, showing that the hour of the day, genotype factors, and interaction between factors, play a role in results (p<0.0001 for each analysis). "#" indicates a significant difference (p<0.05) between Control and Dab mutant files at the same hour of the day. **e** Number of eggs from Dab mutant animals. t-test P=0.0065. ** mean P<0.01 n=three independent experiments carried out with 30 females from *c309,eGFP*/+ and *c309,eGFP*/+;*$Dab^1$*/+ strains. **f** Number of eggs from LpR1 and LpR2 mutants. One Way ANOVA, Tukey post-test P=0.0162, * means P<0.005. n=3 independent experiments carried out with 30 females from *c309,eGFP*/+;*$LpR1^{DF}$*/+ and *c309,eGFP*/+;*$LpR2^{DF}$*/+, and *c309,eGFP* control animals.

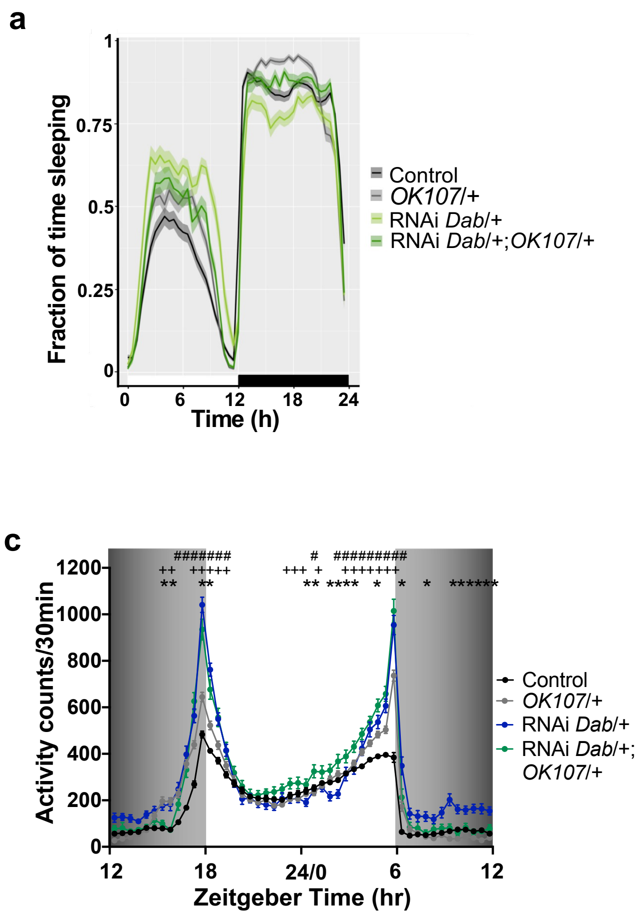


**Fig. S11 Flies knockdown for Dab in the MB presents normal MB associated behaviors. a** Sleep profile in flies expressing an RNAi against Dab in the MB. The lower white left bar represents the hours of the day where flies were exposed to light. Lower black right bar represents the hours of the day were fly were exposed to darkness. **b** Fraction of time sleeping in flies knockdown for Dab in the MB. Two-way ANOVA show that brightness and genotype factors, and also interaction between factors, play a role in results ($p<0.0001$ for each analysis), Tukey post test; * and **, indicates $p<0.05$ and $p<0.01$ between conditions. ns, not significant. **c** Locomotor activity profile throughout the day in flies expressing an RNAi against Dab. Two-way ANOVA, Tukey post test, show that the hour of the day, and genotype factors, and also interaction between factors, play a role in results ($p<0.0001$ for each analysis). "+" indicates significant difference ($p<0.05$) between *OK107*/+ control fly and flies expressing a RNAi against Dab in the MB (RNAi *Dab*/+;*OK107*/+ ), at a given time of the day; "#", significant difference ($p<0.05$) between CS control flies and the knockdown for Dab in the MB at the same hour of the day. "*" indicates significant difference ($p<0.05$) betweeen RNAi *Dab*/+ control fly and flies expressing a RNAi against Dab in the MB at a given time of the day. **a-c** Data expressed as mean ± SEM. Data in **a-c**, from two independent experiments, were from n=32-95 flies studied per genotype. Strain identified as control strain is Canton-S
