# Supplementary material for: Lipophorin receptors regulate mushroom body development and complex behaviors in Drosophila
Source: BMC Biol. 2022 Sep 7;20:198. doi: 10.1186/s12915-022-01393-1 (PMC9454125; doi:10.1186/s12915-022-01393-1)
Supplement: Supplementary file 2 — Additional file 2: Table S1. Phenotypes of flies haploinsufficient or knockout of LpR1 or LpR2 and after the re-expression of LpR2. Table S2. Phenotypes in insertion mutant flies for LpR1 and LpR2 and after the re-expression of LpR2. Table S3. Phenotypes of flies that express RNAi against LpR1 or LpR2 in MB neurons under the control of the c309-Gal4 driver. Table S4. Phenotypes of flies expressing RNAi against LpR1 or LpR2 in MB neurons under the control of the OK107 driver. Table S5. Phenotypes of mutant flies lacking a copy of Dab. Table S6. Phenotypes of flies that express an RNAi against Dab in MB neurons directed by c309-Gal4. Table S7. Phenotypes in the evaluation of genetic interaction between LpR1 and Dab. [file 12915_2022_1393_MOESM2_ESM.pdf]

Table S1. Phenotypes of flies haploinsufficient or knockout of LpR1 or LpR2 and after the re-expression of LpR2. .

	c309,eGFP	*c309,eGFP/+; LpR1^{DF}/+*	*c309,eGFP/+; LpR1^{DF}/LpR1^{DF}*	*c309,eGFP/+; LpR2^{DF}/+*	*c309,eGFP/* LpR2 20Kb BAC; *LpR2^{DF}/+*	*c309,eGFP/+; LpR2^{DF}/LpR2^{DF}*
Normal	40	34	27	43	23	23
Emergence of axons from a β lobe that touch the opposite β lobe			1	2		
merge of β lobes		1	1			1
Mislocalizated α		2				1
Mislocalizated β		1				
Without lobe(s)		2	3	1		2
Short α lobe			1			2
Thin lobe(s)		1	2			2
Total	40	41	35	46	23	31

Table S2. Phenotypes in insertion mutant flies for LpR1 and LpR2 and after the re-expression of LpR2.

	W^{1118}	*LpR1 CRIMIC/+*	*LpR1 CRIMIC/ LpR1 CRIMIC*	*LpR2 CRIMIC/+*	LpR2 20Kb, BAC/+; *LpR2 CRIMIC/+*	*LpR2 CRIMIC/ LpR2 CRIMIC*
Normal	14	44	14	30	12	22
Emergence of axons from a β lobe that touch the opposite β lobe		3	2	1		1
merge of β lobes		7	13	2		2
Mislocalizated α						1
Mislocalizated β		1				
Without lobe(s)		1	1	4		1
Short α lobe		1				
Thin lobe(s)						3
Total	14	57	30	37	12	30

Table S3. . Phenotypes of flies that express RNAi against LpR1 or LpR2 in MB neurons under the control of the c309-Gal4 driver

	c309,eGFP	RNAi *LpR1*/+	*c309,eGFP*/+; RNAi *LpR1*/+	RNAi *LpR2*/+	*c309,eGFP*/+; RNAi *LpR2*/+
Normal	26	15	11	18	20
Emergence of axons from a β lobe that touch the opposite β lobe			7		3
Full or partial merge of β lobes			2		1
Short α lobe					2
Thin lobe(s)			1		
One MB					1
Total	26	15	21	18	27

Table S4. Phenotypes of flies expressing RNAi against LpR1 or LpR2 in MB neurons under the control of the OK107 driver.

	CD8::GFP/+;; *OK107*	RNAi *LpR1*/+	*CD8::GFP*/+; RNAi *LpR1*/+; *OK107*/+	RNAi *LpR2*/+	*CD8::GFP*/+; RNAi *LpR2*/+; *OK107*/+
Normal	25	15	12	18	19
Emergence of axons from a β lobe that touch the opposite β lobe			5		2
Full or partial merge of β lobes			6		1
Short α lobe			1		
Thin lobe(s)					4
Without lobe(s)					3
Total	25	15	24	18	29

Table S5. Phenotypes of mutant flies lacking a copy of Dab.

	c309,eGFP	*c309,eGFP/+;Dab^1/+*
Normal	36	31
Mislocalizated β		3
Split β lobes		1
Total	36	34

Table S6. Phenotypes of flies that express an RNAi against Dab in MB neurons directed by *c309-Gal4*.

	c309,eGFP	RNAi *Dab*/+	*c309,eGFP/+*;RNAi *Dab*/+
Normal	26	18	20
Emergence of axons from a β lobe that touch the opposite β lobe			12
Full or partial merge of β lobes			2
Total	26	18	34

Table S7. Phenotypes in the evaluation of genetic interaction between LpR1 and Dab.

	Dab^1/+	*LpR1 CRIMIC/+*	*LpR1 CRIMIC/Dab^1*
Normal	19	44	13
Emergence of axons from a β lobe that touch the opposite β lobe	1	3	4
merge of β lobes		7	11
Mislocalizated α			
Mislocalizated β		1	
Without lobe(s)		1	
Short α lobe		1	
Thin lobe(s)			
Total	20	57	28
